# Mitochondrial Redox Vulnerabilities in Triple-Negative Breast Cancer: Integrative Perspectives and Emerging Therapeutic Strategies

**DOI:** 10.3390/metabo16010060

**Published:** 2026-01-09

**Authors:** Alfredo Cruz-Gregorio

**Affiliations:** Departamento de Fisiología, Instituto Nacional de Cardiología Ignacio Chávez, Tlalpan, Mexico City 14080, Mexico; alfredo.cruz@cardiologia.org.mx

**Keywords:** triple negative breast cancer, redox state, mitochondria, redox therapeutics, oxidative stress

## Abstract

Breast cancer is a significant public health concern, with triple-negative breast cancer (TNBC) being the most aggressive subtype characterized by considerable heterogeneity and the absence of estrogen receptor (ER), progesterone receptor (PR), and human epidermal growth factor receptor 2 (HER2) expression. Currently, there are no practical alternatives to chemotherapy, which is associated with a poor prognosis. Therefore, developing new treatments for TNBC is an urgent need. Reactive oxygen species (ROS) and redox adaptation play central roles in TNBC biology. Targeting the redox state has emerged as a promising therapeutic approach, as it is vital to the survival of tumors, including TNBC. Although TNBC does not produce high levels of ROS compared to ER- or PR-positive breast cancers, it relies on mitochondria and oxidative phosphorylation (OXPHOS) to sustain ROS production and create an environment conducive to tumor progression. As a result, novel treatments that can modulate redox balance and target organelles essential for redox homeostasis, such as mitochondria, could be promising for TNBC, an area not yet reviewed in the current scientific literature, thus representing a critical gap. This review addresses that gap by synthesizing current evidence on TNBC biology and its connections to redox state and mitochondrial metabolism, with a focus on innovative strategies such as metal-based compounds (e.g., copper, gold), redox nanoparticles that facilitate anticancer drug delivery, mitochondrial-targeted therapies, and immunomodulatory peptides like GK-1. By integrating mechanistic insights into the redox state with emerging therapeutic approaches, I aim to highlight new redox-centered opportunities to improve TNBC treatments. Moreover, this review uniquely integrates mitochondrial metabolism, redox imbalance, and emerging regulated cell-death pathways, including ferroptosis, cuproptosis, and disulfidptosis, within the context of TNBC metabolic heterogeneity, highlighting translational vulnerabilities and subtype-specific therapeutic opportunities.

## 1. Introduction

Triple-negative breast cancer (TNBC) represents the most aggressive subtype of breast cancer and is characterized by rapid progression, early metastasis, and limited therapeutic options. Unlike hormone receptor-positive and human epidermal growth factor receptor 2 (HER2)-amplified tumors, TNBC lacks validated molecular targets, making tumor metabolism and redox biology increasingly relevant as therapeutic entry points.

Accumulating evidence indicates that TNBC cells rely on a highly adaptable redox system to support proliferation, survival under hypoxic stress, and treatment resistance. This adaptation includes increased mitochondrial plasticity, altered oxidative phosphorylation (OXPHOS), and the ability to fine-tune reactive oxygen species (ROS) production to maintain a permissive redox environment.

Importantly, TNBC cells tolerate higher basal ROS levels than other breast cancer subtypes, allowing them to activate pro-survival pathways while remaining vulnerable to additional oxidative stress. This “redox threshold” is emerging as a central vulnerability that can be exploited pharmacologically. Moreover, growing evidence indicates that redox imbalance and metabolic reprogramming are tightly interconnected hallmarks of aggressive cancers, including TNBC, and represent emerging therapeutic entry points [1].

Several reviews have addressed aspects of redox biology in cancer or mitochondrial metabolism in breast cancer [2]. Still, none, to my knowledge, have specifically integrated these domains into TNBC and emerging therapeutic strategies. The existing literature tends to focus either on redox signaling in general oncology or on TNBC metabolism, without exploring targeted redox interventions. This review aims to bridge that gap by critically synthesizing evidence on the interplay between mitochondrial function, redox regulation, and therapeutic approaches in TNBC, highlighting recent advances in treatments such as metal-based compounds (e.g., copper, gold), redox nanoparticles that facilitate the release of anticancer drugs, mitochondrial-targeted therapies, and immunomodulatory peptides, like GK-1, as well as future clinical perspectives. Thus, I integrate current evidence on the redox phenotype of TNBC, highlight mitochondrial vulnerabilities, and discuss therapeutic strategies to exploit redox imbalance by targeting metabolic inhibitors, redox-active compounds, and mitochondrial functions.

**Figure 1:**
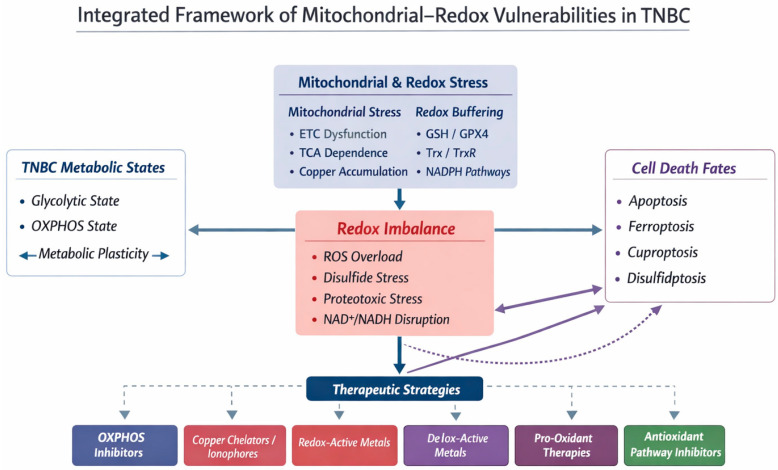
An integrated framework of mitochondrial and redox vulnerabilities in triple-negative breast cancer (TNBC). The schematic summarizes major sources of mitochondrial stress (electron transport chain dysfunction, tricarboxylic acid cycle (TCA)-cycle activity, copper imbalance), redox-regulatory systems (glutathione, thioredoxin, nicotinamide adenine dinucleotide phosphate (NADPH)-dependent pathways), and metabolic plasticity (glycolytic versus oxidative phosphorylation (OXPHOS)-dependent states) in TNBC. These interconnected processes shape susceptibility to distinct regulated cell-death programs, including apoptosis, ferroptosis, cuproptosis, and disulfidptosis, and define vulnerabilities that mitochondria can exploit- and redox-targeted therapeutic strategies. The framework highlights how mitochondrial metabolism and redox balance act as central integrators of TNBC heterogeneity and therapeutic response.

To integrate these concepts, this review is organized around a unifying framework in which mitochondrial metabolic states and redox regulation converge to determine cell-fate decisions in TNBC. I first discuss metabolic plasticity and mitochondrial ROS generation, followed by redox-buffering systems and emerging regulated cell-death pathways. Finally, I examine how these vulnerabilities are therapeutically exploited across preclinical and clinical settings. Thus, I provide a unifying conceptual framework for the diverse mitochondrial and redox-related mechanisms discussed throughout this review, and I present an integrated schematic model (Figure 1) summarizing the major sources of mitochondrial stress, redox buffering systems, metabolic states, cell-death pathways, and therapeutic interventions relevant to TNBC.

## 2. TNBC and Redox State

**Figure 2:**
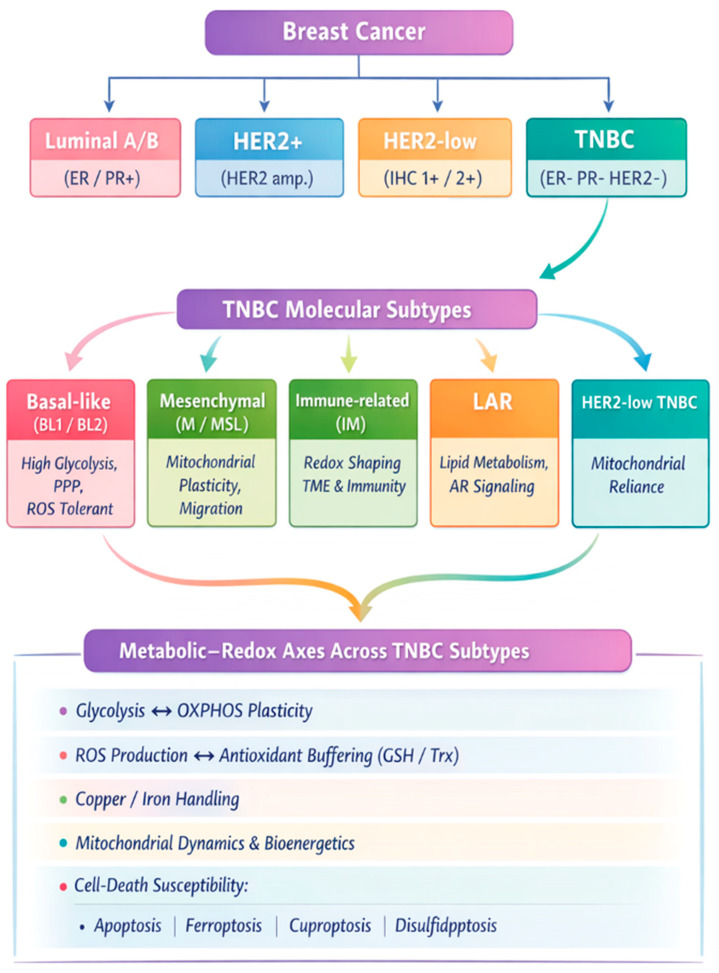
Types of breast cancer and metabolic–redox heterogeneity of triple-negative breast cancer (TNBC). Breast cancer is classified into luminal, human epidermal growth factor receptor 2 (HER2)-positive, HER2-low, and triple-negative subtypes. (OXPHOS), antioxidant capacity, mitochondrial plasticity, and susceptibility to redox-regulated cell-death pathways, which collectively define therapeutic vulnerabilities. TNBC is further subtyped into basal-like (BL) 1 and BL2, immunomodulatory (IM), mesenchymal (M), mesenchymal-like (MSL), and luminal androgen receptor (LAR) subtypes. Each is associated with distinct metabolic programs and redox dependencies. These include differential reliance on glycolysis versus oxidative phosphorylation.

Cancer is characterized by uncontrolled cell proliferation driven in part by metabolic reprogramming that sustains growth and survival. This results from various cellular transformations, some of the most important of which are genetic mutations [3]. These transformations confer immortality and a high capacity for proliferation, as well as the potential to migrate, invade, and colonize other parts of the body, making them among the leading causes of death worldwide [4]. Among the most frequent cancers are breast cancer. Being the second most common cancer worldwide, with 2.33 million cases in 2020, it is known that 1 in 12 women will develop this neoplasia at some point in their life [5]. Breast cancer is subdivided into different types. For instance, progesterone receptor (PR)-positive, estrogen receptor (ER)-positive, HER2-positive, which does not express hormone receptors but overexpresses HER2, and TNBC, which is negative for PR, ER, and HER2 [6]. Moreover, TNBC is a very diverse group of cancers. Lehmann et al. [7] identified six TNBC subtypes, including two basal-like (BL1 and BL2), one immunomodulatory (IM), one mesenchymal (M), one mesenchymal stem-like (MSL), and one luminal androgen receptor (LAR) subtype (Figure 2) [7]. Due to this heterogeneity and the lack of molecular targets, its treatment is a challenge. TNBC is also a highly invasive cancer, which can invade both the brain and visceral organs, with a post-metastasis survival rate of 13.3 months [8]. This is a consequence of its aggressiveness and the limited success of current antineoplastic treatments. Due to the limited therapeutic options for this cancer, it is necessary to search for new therapeutic targets. One possible therapeutic target is the cellular redox state and mitochondrial metabolism in TNBC.

The redox state is defined as the equilibrium between oxidizing molecules, such as ROS, and antioxidants. This homeostasis maintains ROS to serve as second messengers in cellular signaling. Still, if they are not controlled, they can be reactive and oxidize the cell’s biomolecules [9]. Thus, a balance must be maintained between ROS production and antioxidant production to maintain a redox homeostasis. Antioxidants can be classified into two types: enzymatic ones, such as superoxide dismutase (SOD), which catalyzes the dismutation of superoxide (O_2_^−^) into hydrogen peroxide (H_2_O_2_), and catalase (CAT) and glutathione peroxidase (GPx), which convert H_2_O_2_ into water.

On the other hand, non-enzymatic antioxidants, such as ascorbic acid (vitamin C), glutathione (GSH), and tocopherol (vitamin E), donate hydrogen atoms or electrons [10]. When the concentration of ROS in the cell increases due to increased ROS production or decreased antioxidant production, oxidative stress can develop, leading to cellular damage [11]. It should be noted that mitochondria are prominent producers of ROS. In non-transformed cells, OXPHOS releases between 1% and 4% of electrons, thereby inducing the partial reduction of oxygen to O_2_^−^ [10]. However, ROS production in the ETC increases when electrons leak; for instance, in pathological conditions, mitochondrial decoupling can occur, and electron leakage can rise to 24% [12]. Mitochondrial ROS in TNBC arise mainly from electron leakage at complexes I and III. Although the biochemical steps of the electron transport chain have been extensively characterized, the key point for TNBC is that mitochondrial dynamics and metabolic rewiring modulate the efficiency of electron transfer and thereby the rate of ROS generation. This functional coupling is what shapes the redox phenotype of TNBC.

In addition to mitochondria, another important source of ROS is the NADPH oxidase (NOX) system, a multiprotein complex located in the cytoplasm and on cytoplasmic membranes [13]. This system oxidizes nicotinamide adenine dinucleotide phosphate (NADPH) to NADP^+^ and reduces molecular oxygen to O_2_^−^, which is then metabolized to H_2_O_2_ by SOD-1. It should be noted that H_2_O_2,_ in the presence of reduced transition metals such as iron and chromium, generates hydroxyl radicals (^•^OH) via the Fenton and Haber–Weiss reactions, thereby promoting carcinogenesis [14].

In breast cancer, an imbalance in the redox state induced by overproduction of ^•^OH has been observed, causing oxidative stress and ultimately oxidative damage to cellular biomolecules such as DNA [15,16]. This DNA damage induces modifications in purine and pyrimidine bases and alterations in the structure of DNA chains, leading to errors in transcription and replication and thus promoting mutagenesis and carcinogenesis [4]. It is worth noting that in estrogen-positive breast cancers (MCF-7 cells), the levels of DNA damage detected by 8-hydroxy-2-deoxyguanosine (8-OHdG) are 9 times higher than in TNBC (MDA-MB-231cells) [17], and inhibition of estrogen receptor alpha expression significantly activates the nuclear factor erythroid 2-related factor 2 (Nrf2) pathway, reducing estrogen-induced 8-OHdG formation in MCF-7 cells [18]. Thus, the reduction of oxidative DNA damage is associated with Nrf2 activation, a transcription factor induced by oxidative stress. The latter is because Nrf2 activation increases antioxidant enzyme levels, such as catalase, which suppress ROS. Indeed, Glorieux et al. [19] found that catalase overexpression in mammary cancer cells leads to a less aggressive phenotype and increased sensitivity to chemotherapy treatments, such as paclitaxel, etoposide, and arsenic trioxide.

Accumulating evidence indicates that metabolic and redox dependencies in TNBC are not uniform but vary across molecular subtypes. BL1 and BL2 are characterized by high proliferative rates, elevated DNA damage response activity, and strong glycolytic signatures, consistent with increased glucose uptake and reliance on aerobic glycolysis (Table 1) [7,20]. These features suggest a heightened sensitivity to therapies that exacerbate oxidative stress or impair nucleotide synthesis but a relative tolerance to mitochondrial inhibition.

In contrast, M and MSL subtypes exhibit enrichment of epithelial–mesenchymal transition programs, lipid metabolism, and mitochondrial gene expression, indicating a greater dependence on oxidative phosphorylation and mitochondrial function [1,7]. This metabolic profile implies potential vulnerability to mitochondrial disruptors, redox-active metal complexes, or agents targeting mitochondrial ROS homeostasis.

The luminal androgen receptor (LAR) subtype displays distinct metabolic features, including lipid and steroid metabolism and increased mitochondrial activity, accompanied by enhanced antioxidant defenses [7,21]. Such characteristics may confer resistance to pro-oxidant strategies while favoring combination approaches that simultaneously target mitochondrial metabolism and redox buffering systems.

Collectively, these observations support the concept that TNBC redox vulnerabilities are subtype-dependent and underscore the need for stratified therapeutic strategies rather than a uniform redox-targeting approach.

TNBC subtypes display distinct metabolic phenotypes that shape their redox dependencies and therapeutic vulnerabilities. Basal-like tumors often exhibit high proliferative rates and glycolytic flux, whereas mesenchymal and LAR subtypes show greater reliance on mitochondrial metabolism and fatty acid oxidation. Subtype-specific redox adaptations, including variable antioxidant buffering capacity and mitochondrial tolerance to ROS, accompany these differences. Although direct clinical validation remains limited, integrating subtype-specific metabolism with redox vulnerabilities provides a rational framework for therapeutic stratification and combination strategies. Table 2 provides an integrative overview linking TNBC subtypes, metabolic phenotypes, redox features, and therapeutic implications.

Table 2 provides an integrative overview linking TNBC subtypes, metabolic phenotypes, redox features, and therapeutic implications.

## 3. Mitochondrial Metabolism, Redox Balance, and Immune Modulation in the TNBC Tumor Microenvironment

TNBC develops within a metabolically constrained tumor microenvironment characterized by hypoxia, nutrient competition, and oxidative stress. These conditions promote metabolic coupling between tumor cells and stromal components, reshaping mitochondrial activity, redox balance, and metabolite availability. Mitochondrial metabolism in cancer cells not only sustains ATP production but also modulates the extracellular redox tone through ROS release, lactate export, and consumption of glucose and amino acids, thereby influencing immune cell fitness within the tumor niche [22,23,24].

Mitochondrial ROS act as critical signaling molecules in immune cells, but excessive oxidative stress impairs antitumor immunity. Elevated mtROS and limited nutrient availability promote CD8^+^ T-cell exhaustion by disrupting mitochondrial integrity and NAD^+^/NADH balance, while redox stress influences macrophage polarization toward immunosuppressive phenotypes. These mechanisms have been demonstrated across solid tumors, including breast cancer, and are particularly relevant to TNBC, which frequently exhibits hypoxia-driven mitochondrial rewiring and high oxidative burden [25,26,27].

Importantly, mitochondrial redox state also regulates immunogenic versus non-immunogenic cell death. Mitochondria-derived ROS contribute to immunogenic cell death signaling by promoting danger-associated molecular pattern (DAMP) release and immune recognition; however, excessive antioxidant buffering, via glutathione, thioredoxin, or NADPH-generating pathways, may suppress these signals and facilitate immune evasion. Thus, mitochondrial redox adaptations in TNBC may indirectly limit immune surveillance despite enhancing tumor cell survival [28,29]. Together, these observations highlight mitochondrial redox metabolism as a central determinant of both tumor-intrinsic fitness and immune responsiveness within the TNBC microenvironment.

Mitochondrial metabolism and redox balance in TNBC cells exert profound effects on the tumor immune microenvironment. Mitochondrial ROS (mtROS) act not only as intracellular signaling molecules but also as modulators of tumor immunogenicity, influencing antigen presentation, cytokine secretion, and immune checkpoint expression. Excessive mtROS can promote immunogenic cell death characterized by calreticulin exposure, ATP release, and high mobility group box 1 (HMGB1) secretion, thereby enhancing dendritic cell activation and antitumor T-cell responses [28].

Conversely, chronic redox buffering and metabolic rewiring under hypoxic conditions favor immune evasion. Hypoxia-driven mitochondrial adaptations increase NADPH-dependent antioxidant capacity and lactate production, suppressing cytotoxic T-cell function while promoting regulatory T cells and tumor-associated macrophage polarization toward immunosuppressive phenotypes [23,30].

Redox metabolism also directly shapes immune cell function. T-cell activation and effector differentiation depend on tightly regulated mitochondrial ROS signaling, while excessive oxidative stress impairs T-cell persistence and antitumor activity [25]. Collectively, these findings indicate that mitochondrial redox states in TNBC cells and immune infiltrates jointly determine immune surveillance versus immune escape, providing a rationale for combining redox-targeted therapies with immunomodulatory approaches in TNBC.

## 4. TNBC and Its Treatments

TNBC occurs primarily in young, premenopausal women under 40 years of age (15–20%), and the mortality rate is 40% in the first 5 years after diagnosis. Once metastasis is diagnosed, the survival time is 13 months [31]. The treatment recommended for this cancer is surgery, followed by chemotherapy, since this type of cancer does not respond to endocrine therapy or HER2 receptors as the main systemic option [32]. If the tumor is massive, neoadjuvant chemotherapy is recommended to reduce the tumor size. Surgery is then performed, and adjuvant chemotherapy can be continued to eliminate any cancer cells that remain postoperatively [33]. Standard regimens, including taxanes, anthracyclines, and platinum-based agents, have demonstrated efficacy in early and advanced diseases. Still, the prognosis for patients with TNBC remains worse compared to other breast cancer subtypes. More recently, immunotherapies, such as immune checkpoint inhibitors, and targeted agents, such as poly (ADP-ribose) polymerase (PARP) inhibitors, have expanded the therapeutic arsenal. Yet their benefit is restricted to selected patient subsets, often those with BReast CAncer gene 1 (BRCA) mutations or programmed death-Ligand 1 (PD-L1) positivity. Despite these advances, relapse and resistance are common, and the median overall survival in metastatic TNBC is still markedly poor.

Below, I list some chemotherapy drugs used in the treatment of TNBC, such as taxanes. Their mechanism of action is based on inhibition of microtubule depolymerization, which prevents spindle formation and, consequently, cell division [34]. Genetic profiling performed by Lehman et al. [7] showed that microtubule-associated protein (MAP) 2 and genes involved in mitosis or ceramide metabolism in the BL subtype present a high expression. So that this TNBC subtype could be sensitive to antimitotic drugs such as taxanes. In fact, patients with basal TNBC subtypes (BL1 and BL2) had clinical remission rates 4 times higher than those with MSL and LAR subtypes after taxane therapy [35,36]. Anthracyclines such as doxorubicin inhibit type II topoisomerase, a key enzyme that manipulates DNA topology by creating transient double-strand breaks. Unlike taxanes, no significant differences in anthracycline chemotherapy use have been observed among TNBC subtypes. However, the combination of anthracyclines and taxanes varies considerably between subtypes.

Patients with TNBC of the BL1 or MSL subtype have a higher pathological complete response (pCR) rate, whereas patients with LAR and BL2 TNBC subtypes are not sensitive to the combination regimen. Interestingly, the pCR rate in patients with the BL2 subtype was 0% [34]. It should be noted that anthracyclines reduce the risk of death by 38% in patients under 50 years of age and by 20% in patients between 50 and 59 years of age [37]. The latter relates to the cytotoxicity of these drugs; for instance, anthracyclines, such as doxorubicin, have been shown to induce cardiomyocyte toxicity. Fang et al. [38] demonstrated that this compound induces iron accumulation, associated with ROS production and oxidative stress, which in turn leads to ferroptosis-induced cell death [38]. So, while anticancer drugs can treat cancer, they also have side effects such as cytotoxicity in other organs like the heart, which reduces life expectancy, and this is more pronounced in people over 50. Cyclophosphamide, an alkylating agent that blocks DNA replication, together with Docetaxel, an inhibitor of the dynamics of microtubules, essential components of cell division, is the neoadjuvant chemotherapy regimen with the best pCR in patients with TNBC, with a pCR rate of 50%, compared with very low or zero rates in luminal A and type B subtypes. This indicates that TNBC is the most sensitive subtype to this regimen and is therefore considered the prime candidate to benefit from this treatment [39]. Moreover, it is recommended in a combination therapy regimen that reduces recurrence and prolongs disease-free survival (DFS) if it is combined with doxorubicin and is administered as a dose-dense [40]. However, it is essential to note that, while combinations of anticancer agents are effective for treating TNBC, some anticancer agents that could be synergistic fail to achieve this goal. One example is the observation made with Veliparib, a PARP inhibitor, which was thought to increase cell death through synthetic lethality when used with cyclophosphamide; however, this was not observed in a randomized phase II trial [41]. Although a combination therapy regimen is recommended to reduce recurrence and prolong DFS in TNBC, some drugs have side effects, as seen in doxorubicin or platinum agents such as cisplatin, which inhibits DNA synthesis by forming intra- and inter-strand bonds in DNA, but are highly toxic, presenting neurological toxicity (ototoxicity) and nephrotoxicity. It has been observed that the solubility of these compounds is closely related to their toxicity; for example, carboplatin is much more soluble, which confers less nephrotoxicity [42,43]. These therapeutic limitations underscore that conventional strategies primarily target DNA damage while neglecting TNBC’s profound metabolic and redox vulnerabilities. Importantly, several studies suggest that oxidative stress and mitochondrial metabolism are not only central to tumor biology but also shape therapeutic resistance, offering a conceptual bridge between basic mechanisms and translational opportunities. Thus, understanding how redox state and metabolism influence TNBC progression and therapeutic response is essential for developing innovative treatment strategies, and redox therapy is a promising option for TNBC.

It is essential to note that the association between TNBC and low HER2 expression has recently been demonstrated, which is highly relevant to the treatment of this cancer. For instance, antibody drug conjugates (ADCs) have brought renewed attention to HER2-low breast cancer, defined as HER2 IHC 1+ or 2+ (ISH-negative) and historically classified as HER2-negative. Clinically, HER2-low has emerged less as a stable biological subtype and more as a therapeutic category: several ADCs (notably trastuzumab-deruxtecan/Enhertu) demonstrate activity in HER2-low tumors, expanding treatment options beyond classical HER2-positive disease [44]. Moreover, the frequency and clinical impact of HER2-low status in TNBC warrant attention. Grosse et al. [45] recently analyzed HER2-low expression in TNBC and reported that a measurable fraction of TNBC cases fall into the HER2-low category. Their data suggest that HER2-low status may have prognostic implications that vary with histologic subtype and tumor grade. These findings highlight that a non-negligible subset of tumors previously labeled “triple-negative” could be actionable with HER2-directed ADCs or may exhibit different clinical behavior compared with pure HER2-zero tumors. However, several caveats limit immediate translation. First, studies indicate that HER2-low status is heterogeneous and partially assay-dependent: IHC scoring variability and center-to-center differences can reclassify tumors between HER2-zero and HER2-low, complicating patient selection for ADCs. Transcriptomic measures and improved diagnostic pipelines are being developed to refine detection and reduce misclassification [46]. Second, the biological meaning of HER2-low remains contested. Recent systematic reviews suggest that HER2-low is unlikely to represent a unique biologic subtype and instead serves as a therapeutic biomarker for ADC sensitivity; however, differential outcomes and response rates across cohorts imply that tumor context (molecular subtype, microenvironment, prior therapies) modulates the impact of HER2-low expression. Thus, HER2-low in TNBC may identify a subset with distinct translational opportunities but not a monolithic biological entity [47]. For the redox-metabolism axis, the HER2-low discussion intersects two practical points for TNBC therapeutics: (1) reclassification potential—some tumors amenable to HER2-directed ADCs could be diverted from purely redox/metabolic strategies toward ADC-based combinations; and (2) biomarker integration—combining HER2 status (including quantitative measures), metabolic signatures (e.g., OXPHOS vs. glycolysis), and redox markers (nuclear factor erythroid 2-related factor 2 [Nrf2], NAD(P)H quinone oxidoreductase 1 [NQO1], glutathione metabolism) may permit better patient stratification for combinatorial approaches (e.g., ADC + mitochondrial inhibitor or ADC + redox modulator). In short, HER2-low should be explicitly mentioned in any current TNBC treatment framework because it alters the set of realistic clinical options for a subset of patients [45].

## 5. Metabolic and Redox Characteristics of HER2-Low TNBC

Although HER2-low breast cancer has emerged as a clinically relevant subgroup due to its responsiveness to antibody–drug conjugates, direct evidence linking HER2-low status to specific mitochondrial or redox metabolic dependencies remains scarce, particularly within the TNBC subset. Most studies addressing HER2-low disease have focused on pathological classification and therapeutic outcomes rather than on mitochondrial bioenergetics or redox regulation [45,48].

Nevertheless, emerging concepts in TNBC biology suggest that metabolic and redox heterogeneity may intersect with receptor expression states. TNBC tumors exhibit marked metabolic plasticity, ranging from glycolytic phenotypes to OXPHOS-dependent states driven by peroxisome proliferator-activated receptor-gamma coactivator 1-alpha (PGC-1α) signaling, mitochondrial biogenesis, and enhanced antioxidant buffering [49]. Whether HER2-low TNBC preferentially aligns with any of these metabolic programs remains unresolved.

Importantly, it is currently unknown whether HER2-low TNBC displays increased mitochondrial reliance or enhanced antioxidant capacity compared with HER2-zero TNBC, as no systematic transcriptomic, metabolomic, or functional studies have directly addressed this comparison. This represents a critical knowledge gap, particularly given the potential implications for redox-targeted and mitochondrial-directed therapies.

From a translational perspective, HER2-low status should therefore be viewed as a potential stratification variable rather than a defined metabolic determinant. Future studies integrating HER2-low classification with metabolic profiling, redox gene signatures, and functional sensitivity to mitochondrial stressors will be required to determine whether HER2-low TNBC constitutes a distinct redox-metabolic vulnerability class or merely reflects therapeutic eligibility for ADCs. Collectively, these observations indicate that TNBC metabolic states are not fixed but dynamically adapt to microenvironmental and therapeutic pressures. Such metabolic plasticity has profound consequences for mitochondrial function and redox balance, which in turn shape tumor survival strategies and therapeutic susceptibility. These adaptive redox–mitochondrial programs are discussed in the following sections.

Beyond membrane-associated HER2 expression, several studies have reported the presence of HER2 in the nucleus of breast cancer cells, where it can function as a transcriptional co-regulator involved in cell proliferation and survival. Nuclear HER2 has been primarily described in HER2-amplified and hormone receptor–positive breast cancers, while its presence in TNBC appears to be infrequent and heterogeneous.

At present, there is limited direct evidence demonstrating a consistent or functionally relevant pool of nuclear HER2 specifically in HER2-low TNBC. Consequently, therapeutic strategies directly targeting nuclear HER2 remain largely unexplored in this subtype. However, the emerging recognition of HER2-low status as a biologically distinct entity raises the possibility that non-canonical HER2 signaling, including subcellular localization dynamics, may contribute to tumor heterogeneity and therapeutic response. Further investigation is required to determine whether nuclear HER2 plays a meaningful role in HER2-low TNBC biology and whether it could be exploited therapeutically, either indirectly through antibody–drug conjugates or via modulation of downstream transcriptional programs.

## 6. Hypoxia-Inducible Transcription Factor (HIF) and Redox State in TNBC

TNBC tumors are highly hypoxic and exhibit inflammation, which is associated with elevated ROS production and oxidative stress [50]. Under hypoxic conditions, HIF is activated and translocates to the nucleus, where it forms a heterodimer with HIF-β, promoting the expression of genes that favor the conversion of oxidative metabolism to glycolysis, cell proliferation, and survival by regulating signaling pathways such as phosphoinositide 3-kinase (PI3K)/Akt/ mammalian target of rapamycin (mTOR) [51]. This reprogramming metabolic overexpresses glucose transporters such as glucose transporter 1, increasing glucose internalization and its entry into glycolysis [1]. Thus, TNBC cells frequently exhibit enhanced glucose uptake and a pronounced reliance on aerobic glycolysis, consistent with a Warburg-like metabolic phenotype. This glycolytic dependency is supported by overexpression and hyperactivation of key glucose transporters and glycolytic enzymes, including glucose transporter type 1 (GLUT1), hexokinase 2 (HK2), phosphofructokinase-1 (PFK1), and lactate dehydrogenase A (LDHA), which collectively sustain rapid ATP production and provide metabolic intermediates for biosynthetic and redox-supporting pathways [52,53,54]. In TNBC, elevated glycolytic flux has been linked not only to proliferation and survival but also to redox homeostasis, as glycolysis feeds NADPH-producing pathways such as the pentose phosphate pathway, thereby supporting antioxidant defenses [55]. Consequently, pharmacological targeting of glycolytic enzymes or glucose transporters has emerged as a complementary strategy to mitochondrial inhibition, particularly in glycolysis-dominant TNBC subsets, where mitochondrial targeting alone may be insufficient due to metabolic compensation. On the other hand, ROS can inactivate prolyl hydrolases (HIF antagonists), favoring the activation of HIF-1α. In relation to the latter, HIF-1α is present in 80% of TNBC cases, promoting glycolytic metabolism and disease progression [50,56].

Interestingly, mitochondria have a key role in TNBC chemoresistance. For instance, Evans et al. [57], report that OXPHOS is upregulated in TNBC due to the overexpression of genes encoding ETC complex I. Moreover, these researchers performed RNA sequencing of biopsies from untreated TNBC patients, finding overexpression of mitochondrial genes encoding complex I subunits. The hypothesis that TNBC is OXPHOS-dependent is supported by another study by Winter et al. [58], which used MDA-MB-231 and SUM159-PT TNBC cell lines treated with neoadjuvant chemotherapy, epirubicin and cyclophosphamide, and then with paclitaxel, for a total of 18 weeks. This group demonstrated that the resulting chemo-persistent cell lines were more proliferative; however, the persistent MDA-MB-231 cells became less sensitive to chemotherapeutic drugs than SUM159-PT. The reduction in sensitivity to chemotherapy of the MDA-MB-231 persistent cells was associated with increased OXPHOS, indicating that OXPHOS is upregulated during chemoresistance. However, decreasing OXPHOS by inhibiting pyruvate entry into mitochondria resensitized persistent cells to antineoplastic drugs. Thus, the authors suggest that targeting OXPHOS via pyruvate metabolism can overcome mitochondrial adaptation of chemo-persistent TNBC. Together, these studies indicate that OXPHOS upregulation is a hallmark of metabolic adaptation in chemo-persistent TNBC.

The latter reports highlight the remarkable metabolic heterogeneity of TNBC, ranging from a strong dependence on aerobic glycolysis (“Warburg phenotype”) to an OXPHOS-based phenotype, particularly in mesenchymal and BL2 subtypes. That is, a large number of TNBC tumors present the so-called “Warburg phenotype,” characterized by elevated glucose consumption and lactate production even under normoxic conditions. This is primarily driven by MYC amplification, HIF-1α stabilization, and the overexpression of enzymes such as pyruvate kinase M2 (PKM2) and lactate dehydrogenase A (LDHA), which confer proliferative, invasive, and immune evasion advantages [59]. However, several studies have shown that specific TNBC subtypes, particularly mesenchymal and BL2, exhibit a preference for OXPHOS, associated with PGC-1α activation, increased mitochondrial biogenesis, and reduced glycolytic gene expression [60]. These OXPHOS-dependent cells tend to show a more quiescent state and a stem cell phenotype, which translates into greater therapeutic resistance and an enhanced capacity to adapt to nutritional or pharmacological stress. It is important to note that metabolic plasticity in TNBC enables a dynamic transition between glycolysis and OXPHOS, depending on the tumor microenvironment: while hypoxic zones favor glycolysis, well-oxygenated niches support mitochondrial respiration. In addition, cancer-associated fibroblasts can transfer lactate or fatty acids that fuel the oxidative metabolism of tumor cells [61].

Consequently, the metabolic diversity of TNBC not only reflects intrinsic differences between subtypes, such as increased glycolysis in BL 1 versus oxidative dependence in mesenchymal/BL2, but also has profound therapeutic implications, as blocking one of these metabolic programs in isolation may be insufficient. Hence, combined inhibition of glycolysis and OXPHOS, together with individual metabolic profiling, is currently being explored as an emerging strategy to improve clinical response in patients with TNBC. Despite these advances, essential limitations and controversies persist in TNBC metabolism research. As indicated above, although a dichotomy between glycolytic and OXPHOS phenotypes has been described, most tumors exhibit a highly plastic state, making it difficult to establish universal metabolic markers and complicating the clinical stratification of patients [60]. Furthermore, there is debate about the actual relevance of “metabolic dependence” in in vitro models versus in vivo tumor behavior, where the tumor microenvironment, nutrient availability, and interactions with stromal cells decisively influence metabolism [61]. Another challenge is that many of the therapies targeting glycolysis or OXPHOS have shown preclinical efficacy. Still, their translation into clinical studies is limited by systemic toxicity, metabolic pathway redundancy, and tumor cells’ ability to rapidly adapt to inhibition of a single metabolic axis [62]. Furthermore, there is no consensus on which TNBC subtypes are intrinsically more dependent on OXPHOS or glycolysis, as studies across different cohorts and analysis platforms have yielded divergent results [59]. Finally, a point of contention is the role of cancer stem cells, which appear to be more dependent on OXPHOS, in therapeutic failure. However, it is unclear whether this characteristic is universal or specific to particular tumor niches. Together, these limitations underscore the need for integrative approaches that combine transcriptomic and metabolomic analyses with functional studies in preclinical and clinical models to more precisely define the metabolic landscape of TNBC and its therapeutic implications.

The debate over glycolysis versus OXPHOS dependence is not merely academic but has therapeutic implications. Evidence for glycolysis dependence is most substantial in MYC-amplified tumors, where HIF-1α stabilization drives glucose addiction, whereas OXPHOS reliance appears more consistent in chemoresistant or mesenchymal-like subtypes. Rather than favoring one side, the weight of the evidence suggests that metabolic plasticity itself is the defining hallmark of TNBC, complicating the identification of universal biomarkers and therapeutic targets. Clinically, this implies that single-pathway inhibition is unlikely to succeed, and that combinatorial strategies guided by metabolic profiling may be necessary.

Despite extensive investigation, the relative contribution of glycolysis versus OXPHOS in TNBC remains controversial. Multiple studies describe TNBC as predominantly glycolytic, characterized by high glucose uptake, elevated expression of glycolytic enzymes, and reliance on aerobic glycolysis. In contrast, accumulating evidence indicates that a subset of TNBC tumors and cell lines display significant mitochondrial respiration, enhanced tricarboxylic acid (TCA) cycle activity, and sensitivity to OXPHOS inhibition.

These apparently conflicting observations likely reflect differences in experimental models, nutrient availability, oxygen tension, and, critically, intrinsic TNBC subtype heterogeneity. Two-dimensional cultures often exaggerate glycolytic phenotypes, whereas three-dimensional systems, patient-derived xenografts, and in vivo tumors more frequently reveal mitochondrial dependency. Rather than representing mutually exclusive metabolic states, glycolysis and OXPHOS appear to coexist as interchangeable programs that TNBC cells dynamically engage in response to microenvironmental and therapeutic pressures.

A comparative overview of key studies supporting glycolytic versus OXPHOS-dependent phenotypes in TNBC is summarized in Table 3, highlighting unresolved gaps and model-dependent discrepancies that are directly relevant for metabolic and redox-targeted therapeutic strategies.

Because metabolic rewiring under hypoxia requires robust antioxidant defenses, the HIF pathway is functionally intertwined with the Kelch-like ECH-associated protein 1 (KEAP1)/erythroid 2-related factor 2 (Nrf2) axis. In the next section, I examine how Nrf2-mediated antioxidant responses complement HIF-driven metabolic adaptations, together shaping TNBC redox homeostasis and therapy resistance.

## 7. KEAP1/Nrf2 Pathway in TNBC

**Figure 3:**
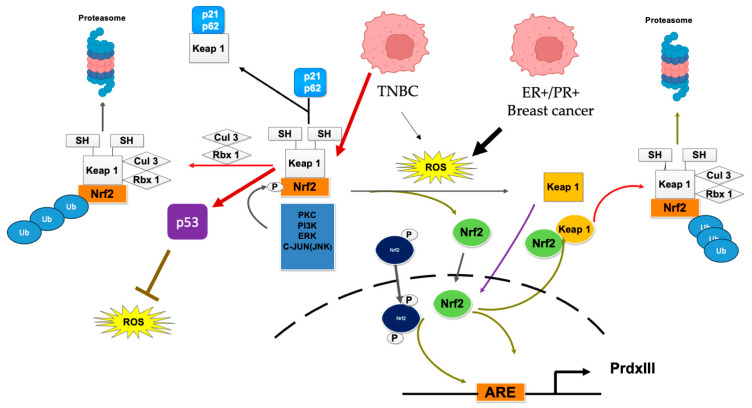
Keap1/Nrf2 pathway in breast cancer. Keap1 is the negative regulator of Nrf2, degrading it under homeostatic conditions. In oxidative stress conditions, Nrf2 is released from Keap1 due to oxidation of Keap1 cysteines that induce conformational changes in Keap1. Then, Nrf2 translocates to the nucleus to activate ARE-responsive genes, involved in the response to oxidative stress or detoxifying activity. When cells reach redox homeostasis, cysteine in Keap1 is reduced, causing Keap1 to translocate to the nucleus, where it binds Nrf2 and escorts it to the cytosol, where Nrf2 is degraded by the proteasome. Nrf2 can be activated by phosphorylation induced by cellular kinases, allowing its release and nuclear translocation, or by p21 and p62 proteins. ER+/PR+ breast cancers produce more ROS than TNBC breast cancers, which activates Nrf2 and the antioxidant response. Conversely, TNBC breast cancers exhibit elevated Keap1 levels, suggesting that Nrf2 activation is not required and is compensated for by p53, which promotes the antioxidant response.

Increased ROS activates Nrf2 as one of the initial mechanisms in response to oxidizing conditions. This is because Nrf2 is a transcription factor that induces the transcription of antioxidant enzymes, such as SOD, CAT, and GPx. Additionally, it induces the transcription of enzymes that synthesize scavenging antioxidants, including GSH. By causing this whole battery of antioxidants, ROS are reduced, and oxidative conditions are prevented, which is associated with DNA damage and the induction of several mutations related to carcinogenesis [63,64]. Thus, Nrf2 suppresses carcinogenesis, especially in its early stages, by maintaining cellular redox homeostasis. In brief, it has been demonstrated that under normal conditions, Nrf2 is negatively regulated by Keap1, which serves as a bridge between Nrf2 and the cullin3 (Cul3)-Rbx1 complex. This E3 ubiquitin ligase conjugates ubiquitin proteins to Nrf2, inducing Nrf2 degradation through the proteasome [65]. However, when cells increase ROS production, Keap1 is oxidized at the thiol groups of its cysteines 226, 613, and 624, which promotes a conformational change in Keap1, allowing the release of Nrf2 (Figure 3). Nrf2 subsequently translocates to the nucleus, where it associates with the antioxidant response element (ARE) thanks to the presence of a DNA-binding leucine zipper (bZip) [66]. Thus, Nrf2 activates many genes ininvolved to ROS reduction, serving as a master transcription factor for maintaining redox homeostasis [67]. Once the redox state is achieved, Keap1 cysteine residues are reduced, allowing Keap1 to translocate to the nucleus and export Nrf2 to the cytosol, where it is ubiquitinated and degraded via the proteasome (Figure 3).

Beyond ROS-mediated activation, Nrf2 can also be activated by hypermethylation of the Keap1 promoter, where Keap1 is not transcribed, thereby inducing Nrf2 activation through epigenetic repression of Keap1 [68]. Another mechanism of Nrf2 activation involves the displacement of Keap1 by the sequestosome (p62) or p21 within the Keap1–Nrf2 complex, allowing the release of Nrf2 [69,70]. Nrf2 can be phosphorylated by several kinases, such as PI3K, protein kinase C (PKC), c-Jun N-terminal kinase (JNK), and extracellular signal-regulated kinases (ERKs), inducing its dissociation from Keap1 and its translocation to the nucleus [71]. ROS induces actin filament depolymerization, which induces the Nrf2-Keap1 complex destabilization, allowing Nrf2 to detach from Keap1 and actin filaments, permitting Nrf2 release and translocation to the nucleus (Figure 3) [72]. The latter mechanisms demonstrate that Nrf2, as a master transcription factor of oxidative stress, functions redundantly in activating the antioxidant machinery through multiple pathways, thereby reducing oxidative stress. It is essential to note that these processes depend on the cellular mechanisms that are carried out at each specific time and space, defining the cellular destiny. Although Nrf2 activation has anticancer properties, it also has malignant side effects. For example, its chronic activation can overexpress antioxidant and detoxifying enzymes that eliminate ROS and xenobiotics, including antineoplastic drugs, thereby favoring therapy resistance and tumor promotion and progression [73,74]. That’s why this molecule is associated with poor prognoses in certain cancers, necessitating Nrf2 deactivation to reduce or eliminate cancerous cells [75].

It has been observed that ER+/PR+ and TNBC breast cancers increase ROS production; however, ER+/PR+ breast cancers produce more ROS than TNBC, mainly because the latter lack estrogen receptors [76]. This increase in ROS induces DNA damage, which was demonstrated by Karihtala et al. [16]. This group used immunostaining studies on ER+/PR+ and TNBC breast cancer cell samples, which showed 8-OhdG levels of 73.2% in ER+/PR+ cells. In contrast, in TNBC cells, 8-OhdG levels reached only 37.1%, supporting the evidence that estrogens promote ROS production [16]. This difference in ROS levels can be associated with the Keap1/Nrf2 signaling pathway. In TNBC, Keap1 has been reported to be highly expressed, suggesting that the activation of the antioxidant system is not required, as estrogen-mediated oxidative stress is not induced [16]. Nevertheless, Pereira et al. [77] performed three-dimensional (3D) cultures of TNBC cells and observed that ROS activate Nrf2 and p53 independently and simultaneously. In their study, they showed that each compensates for the underexpression of the other; that is, if p53 is underexpressed, Nrf2 increases, and vice versa. This indicates that, in Keap1 overexpression associated with Nrf2 deficiency, Nrf2 signaling is dampened by compensatory increases in p53. Thus, cancer cells adapt to conditions of oxidative stress, in which one transcription factor, such as Nrf2, is compensated for by another, such as p53, highlighting adaptive redundancy in TNBC oxidative stress responses. Together, these findings suggest that Nrf2 plays a context-dependent role in TNBC.

Since Nrf2 upregulates mitochondrial antioxidant enzymes such as Mn-SOD, its activity directly influences mitochondrial redox homeostasis. The following section examines how Mn-SOD (SOD2) contributes to TNBC progression and its potential as a therapeutic vulnerability.

## 8. Manganese Superoxide Dismutase (Mn-SOD or SOD2)

SOD2 is a mitochondrial enzyme that catalyzes the dismutation of O_2_^−^ into H_2_O_2_ and O_2_, thereby constituting a central element of the cellular antioxidant system. Structurally, SOD2 is a 96 kDa homotetramer with a manganese catalytic center; it is synthesized in the cytosol and subsequently transported to the mitochondrial matrix [78]. Overexpression of MnSOD increases H_2_O_2_ production. This increase induces the oxidation of the tumor suppressor phosphatase and tensin homologue (PTEN), activates AKT, and initiates a signaling cascade that promotes cell proliferation, growth, and survival [79]. In healthy tissue, MnSOD levels are tightly regulated to prevent H_2_O_2_ overproduction; however, studies show that in various tumors, the genes encoding MnSOD are overexpressed, leading to increased MnSOD levels [80,81].

Al Haq et al. [82] reported the presence of multiple copies of the malignant neoplasia T-cell oncoprotein 1 (MCT-1) in TNBC cells. This overexpression increased the amount of the transcription factor Nrf2, which induces MnSOD transcription, promoting the overproduction of ROS and oxidative stress, both of which are associated with tumor proliferation and carcinogenesis. Therefore, the induction of MnSOD as a pro-oxidant is correlated with a poor prognosis and more aggressive cancer, whereas silencing MnSOD inhibits ROS production and the development and invasion of cancer cells [82]. From the above, it is suggested that we delve deeper into the role of oxidative stress in TNBC, since although ROS are not produced through hormonal mechanisms, they can be generated by the overexpression of MCT-1. This highlights the paradoxical nature of Mn-SOD in TNBC. While classically considered a protective antioxidant enzyme, in this context, it appears to act as a pro-oxidant, maintaining redox imbalance and promoting tumor progression. However, whether Mn-SOD consistently represents a poor prognostic marker across all TNBC subtypes remains unclear. Some evidence suggests that its role may depend on tumor stage, the degree of metabolic heterogeneity, and compensatory pathways such as Nrf2 or p53. The open question is whether direct action on Mn-SOD would be therapeutically advantageous or whether its inhibition could exacerbate genomic instability in normal cells. These controversies underscore the need for functional studies that integrate Mn-SOD activity with the metabolic subtype profile and redox adaptation in TNBC.

Given the central role of Mn-SOD in shaping mitochondrial ROS levels, redox therapies that manipulate glutathione metabolism, NADPH oxidases, or mitochondrial function may exploit this vulnerability. The following section examines current and emerging treatment strategies that specifically target redox state and mitochondria in TNBC.

## 9. Redox Currencies and Metabolic Cofactors Shaping Mitochondrial Vulnerability in TNBC

Beyond ROS themselves, mitochondrial vulnerability in TNBC is critically governed by intracellular redox currencies, particularly the balance of NAD^+^/NADH and NADPH, which integrate metabolic flux, antioxidant capacity, and cell-death susceptibility. NAD^+^ availability sustains oxidative metabolism, supports mitochondrial respiration, and regulates stress responses through sirtuins and PARP-dependent signaling, whereas NADPH fuels reductive biosynthesis and antioxidant systems, including glutathione and thioredoxin pathways [83].

In TNBC, enhanced flux through NADPH-generating pathways—such as the pentose phosphate pathway (PPP), malic enzyme 1 (ME1), and cytosolic isocitrate dehydrogenase (IDH1)—has been associated with increased redox buffering capacity and resistance to oxidative stress. This metabolic configuration supports detoxification of lipid peroxides and mitochondrial ROS, thereby limiting susceptibility to ferroptosis and other oxidative cell-death programs [55,84]. Importantly, elevated NADPH availability can counteract the pro-oxidant effects of redox-targeted therapies, including mitochondrial disruptors and ROS-inducing agents.

GSH and thioredoxin (Trx) systems represent the major downstream effectors of NADPH-dependent redox control. Upregulation of SLC7A11-mediated cystine import, GSH synthesis, GPX4 activity, and Trx/TrxR signaling has been observed in aggressive breast cancer phenotypes, contributing to mitochondrial protection and therapeutic resistance [85]. These systems not only regulate ferroptosis sensitivity but also influence mitochondrial proteostasis and redox signaling, intersecting with emerging forms of cell death such as cuproptosis and disulfide stress–induced cytotoxicity.

Collectively, these findings indicate that TNBC vulnerability to mitochondrial and redox-targeted therapies is not dictated by ROS levels alone, but by the integrated capacity of NAD^+^- and NADPH-dependent buffering systems. Therapeutic strategies that simultaneously disrupt mitochondrial function and constrain redox currencies—such as combining OXPHOS inhibition with blockade of NADPH production or thiol metabolism—may therefore be required to overcome metabolic adaptation and resistance.

## 10. Mitochondrial Dynamics and Mitophagy as Regulators of Redox Adaptation in TNBC

Mitochondrial dynamics, encompassing fission and fusion processes, together with selective mitochondrial turnover via mitophagy, play a critical role in shaping redox homeostasis and therapeutic resistance in TNBC. Fission, primarily mediated by dynamin-related protein 1 (DRP1), promotes mitochondrial fragmentation, facilitating metabolic reprogramming and adaptation to oxidative stress, whereas fusion, regulated by mitofusins (MFN1/2) and optic atrophy protein 1 (OPA1), supports mitochondrial integrity and efficient oxidative phosphorylation [86].

In breast cancer, including TNBC, elevated DRP1 activity has been associated with increased mitochondrial ROS tolerance, enhanced glycolytic flexibility, and resistance to chemotherapeutic stress. Conversely, enforced mitochondrial fusion has been linked to OXPHOS dependency and increased susceptibility to mitochondrial perturbation [87]. These dynamics allow TNBC cells to adapt rapidly to redox stress induced by therapies targeting mitochondrial metabolism.

Mitophagy constitutes a complementary survival mechanism by selectively eliminating damaged, ROS-producing mitochondria. The PINK1 pathway is the best-characterized mitophagy axis and has been implicated in limiting oxidative stress accumulation and preserving bioenergetic fitness in cancer cells under therapeutic pressure [88]. In TNBC models, activation of mitophagy has been shown to attenuate mitochondrial ROS accumulation and reduce sensitivity to mitochondrial inhibitors and pro-oxidant therapies [89,90].

Importantly, mitochondrial dynamics and mitophagy intersect with regulated cell-death pathways. Excessive mitochondrial fission and impaired mitophagy can amplify mitochondrial ROS, promoting apoptosis and ferroptosis, whereas efficient mitophagic clearance may buffer against oxidative damage and support resistance to redox-based therapies. These adaptive mechanisms therefore represent double-edged regulators of mitochondrial vulnerability in TNBC.

Collectively, these findings indicate that mitochondrial dynamics and mitophagy are integral components of the redox adaptive landscape in TNBC. Therapeutic strategies that disrupt mitochondrial metabolism without simultaneously limiting fission-driven adaptation or mitophagy-mediated quality control may be insufficient. Targeting DRP1 activity or mitophagy pathways in combination with redox or mitochondrial inhibitors may enhance therapeutic efficacy and limit resistance.

## 11. Treatments for TNBC Associated with Redox State and Mitochondria

### 11.1. Drugs Targeting Glutathione Metabolism, NADPH Oxidase Inhibitors, or Mitochondrial-Targeted Therapies

Within the mitochondrial–redox framework outlined above, several therapeutic strategies aim to exploit metabolic stress, redox imbalance, or impaired mitochondrial adaptability in TNBC.

For clarity, redox- and mitochondria-targeted strategies in TNBC are organized below according to their primary mechanism of action and cellular compartment, rather than as parallel therapeutic approaches.

**Figure 4:**
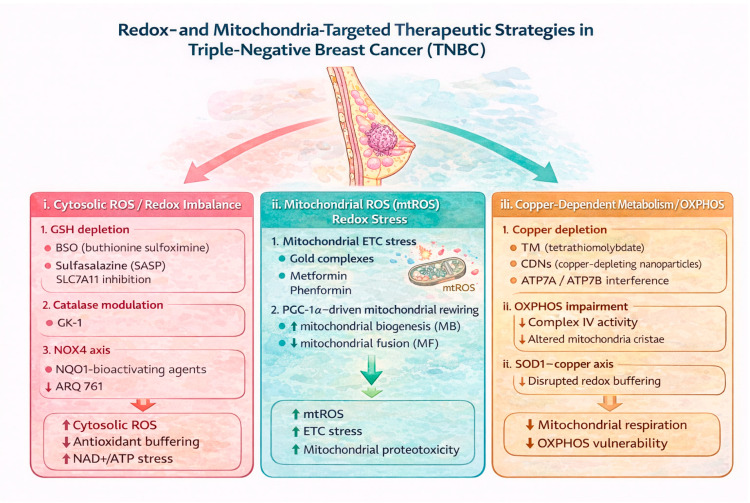
Redox- and mitochondria-targeted therapeutic strategies in triple-negative breast cancer (TNBC). Mechanistic organization of redox- and mitochondria-targeted strategies in TNBC. Therapeutic approaches are grouped according to their primary site of action and redox mechanism, including cytosolic antioxidant depletion, mitochondrial redox stress and mtROS modulation, and copper-dependent OXPHOS vulnerability. Multiple redox-based therapeutic approaches have been proposed for TNBC, targeting distinct but interconnected redox and metabolic vulnerabilities. These strategies can be broadly grouped into (i) cytosolic redox modulation, including glutathione (GSH) depletion, catalase inhibition, NOX4-dependent ROS signaling, and NQO1-mediated redox cycling; (ii) mitochondrial stress and mtROS induction through disruption of the electron transport chain, inhibition of mitochondrial complex I (e.g., metformin, phenformin), and mitochondrial remodeling linked to PGC-1α activity; and (iii) copper-dependent metabolic targeting, including copper depletion strategies (tetrathiomolybdate, copper-depleting nanoparticles), impaired copper transport (ATP7A/ATP7B), and OXPHOS dysfunction. Together, these approaches highlight the diversity of redox and mitochondrial vulnerabilities exploitable in TNBC, emphasizing molecular mechanisms rather than therapeutic endpoints. SASP, sulfasalazine; BSO, buthionine sulfoximine; GSH, glutathione; ROS, reactive oxygen species; mtROS, mitochondrial ROS; NOX4, NADPH oxidase 4; NQO1, NAD(P)H:quinone oxidoreductase 1; PGC-1α, peroxisome proliferator-activated receptor gamma coactivator 1-alpha; MB, mitochondrial biogenesis; MF, mitochondrial fusion; TM, tetrathiomolybdate; CDN, copper-depleting nanoparticle; OXPHOS, oxidative phosphorylation.

Based on these vulnerabilities, multiple therapeutic strategies have been proposed to perturb redox homeostasis and mitochondrial function in cancer cells [91,92,93]. For instance, inhibition of GSH biosynthesis has emerged as a promising strategy in TNBC, as these cells depend heavily on this antioxidant to counteract the oxidative stress associated with their high proliferative rate. For instance, Betty et al. [94] employed mass spectrometry-based metabolomics to identify metabolic dependencies in TNBC, finding that GSH levels were significantly lower in TNBC cell lines compared to non-transformed mammary epithelial cells. Two metabolic subtypes within TNBC were identified, correlating with markers of BL and non-BL status. Non-BL cell lines showed increased sensitivity to pharmacological inhibition of GSH biosynthesis. This effect was rescued by the antioxidant N-acetylcysteine, indicating a dependence on GSH production to suppress ROS and support tumor survival. Clinically, patients whose tumors expressed elevated levels of γ-glutamylcysteine ligase, the rate-limiting enzyme in glutathione biosynthesis, had significantly worse survival outcomes. These mechanistic categories are summarized in Figure 4.

Agents that limit the availability of cysteine as GSH precursors, such as cystine/glutamate antiporter xCT, sulfasalazine (SASP), and erastin or BSO, were found to potentiate both GSH depletion and γ-glutamylcysteine ligase inhibitor-mediated TNBC cell death in vitro. Significantly, this approach also suppressed GSH levels and TNBC xenograft growth in in vivo models. Together, these findings support targeting the GSH biosynthetic pathway as a therapeutic strategy in TNBC and point to the non-basal-like subtype as the most likely to respond. Note that ferroptosis inducers, such as erastin, exacerbate GSH depletion, thereby promoting iron accumulation and lipid peroxidation, which contribute to ferroptosis. This iron-dependent cell death could be particularly effective in TNBC (Table 4 and Figure 4) [95,96]. Thus, manipulating GSH homeostasis and ferroptosis regulators opens a unique therapeutic window for treating TNBC.

Indeed, managing the redox state in TNBC has emerged as a promising target, with strategies ranging from inhibiting GSH metabolism to advanced mitochondrial therapies, including targeting NADPH oxidases (NOX). For example, overexpression of NOX4 (a major mitochondrial ROS producer) in TNBC lines has been shown to decrease cell migration, invasiveness, and proliferation [97]. Interestingly, NOX4 overexpression enhanced PGC1α, inducing mitochondrial biogenesis and leading to increased mitochondrial mass and elongated mitochondrial morphology, which is related to mitochondrial fusion. Conversely, NOX attenuated Drp1-mediated mitochondrial fission [97]. Moreover, studies in murine TNBC models indicate that NOX4 loss worsens tumor prognosis, and in vitro silencing of NOX4 enhances proliferation, colony formation, and migration. Authors of this work related the mechanism to an increase in glycolytic metabolism, lower infiltration of CD8^+^ lymphocytes, and a decreased response to immunotherapy; in contrast, NOX4 overactivation improved survival and demonstrated an anti-tumor immunity [98]. These findings highlight the function of NOX4 in TNBC, suggesting NOX4 as a possible therapeutic target.

TCA cycle dehydrogenases represent an additional, although less explored, layer of metabolic regulation in TNBC. Although oncogenic mutations in isocitrate dehydrogenase (IDH1/IDH2) are well established in gliomas and acute myeloid leukemia, such mutations are rare in breast cancer, including TNBC, and therefore are unlikely to constitute a dominant bioenergetic driver in this disease [107,108]. Nevertheless, wild-type IDH enzymes contribute substantially to cellular redox homeostasis through the generation of NADPH, which supports GSH recycling and resistance to oxidative stress [109,110]. In breast cancer models, NADPH-producing pathways, including IDH1/IDH2 activity, have been implicated in buffering mitochondrial and cytosolic ROS rather than directly enhancing ATP production [85,111]. Thus, modulation of IDH-dependent redox balance may indirectly influence TNBC sensitivity to redox-based or mitochondria-targeted therapies, even in the absence of oncogenic IDH mutations.

### 11.2. Mitochondrial Redox Stress and mtROS Modulation

Regarding dietary antioxidants, they have shown benefits in preclinical models. For instance, a combination of three polyphenols: quercetin, curcumin, and berberine, has been demonstrated to reduce epithelial-mesenchymal transition (EMT) proteins in TNBC cells, inducing pro-apoptotic, anti-proliferative, anti-migratory, and anti-metastatic effects [112]. However, their impact on humans is variable due to low bioavailability and potential protective effects on tumor cells. Mitochondrial therapies, such as metformin, have been shown to reduce proliferation, invasion, migration, and adhesion in the MDA-MB-231 TNBC cell line, thereby inducing apoptosis. Moreover, in vivo models showed that post-metformin treatment induced tumor reduction in both size and weight, and there was a decrease in lymph node plasma cell proliferation and tumor angiogenesis [113]. Even phenformin significantly inhibited TNBC tumors induced by MDAMB231, showing a greater efficacy than metformin without murine toxicity [114]. In parallel, mitochondrial-targeted prodrugs such as MitoTam [115], CPI-613 [99], and mitochondria-targeted antioxidants such as MitoQ [100] offer more selective ways to modulate mitochondrial reactive oxygen species (mtROS) and alter TCA metabolism. In brief, Rohlenova et al. [115] demonstrated that selective disruption of respiratory supercomplexes by MitoTam more efficiently suppresses Her2^high^ tumors than TNBC, highlighting TNBC’s lower sensitivity to this strategy due to its high metabolic plasticity. Along these lines, Shen et al. [99] demonstrated that dihydrolipoamide S-succinyltransferase (DLST, a key TCA enzyme) inhibition suppresses growth and induces cell death in subsets of human TNBC cell lines. This highlights that mitochondrial inhibitors, such as CPI-613 (α-ketoglutarate dehydrogenase complex inhibitor), can also target and exploit this vulnerability to increase metabolic stress in TNBC. Finally, Capeloa et al. [100] demonstrated that the mitochondrial antioxidant MitoQ prevents metastasis in MDA-MB-231-induced TNBC xenograft, confirming that mitochondrial redox manipulation may have a therapeutic effect in this particularly aggressive subtype. Together, these studies suggest that while Her2^high^ tumors respond best to supercomplex disruption, TNBC requires combined strategies that integrate metabolic inhibitors, such as CPI-613, and redox modulators, such as MitoQ, to overcome its heterogeneity and therapeutic resistance. For instance, the latter approach can be complemented by innovations in redox nanomedicine, such as diselenide prodrugs (FA-SeSe-NPs), which induce ROS accumulation. It has been demonstrated that these ROS generate selenium-free radicals, which in turn increase ROS that react with GSH to form S-Se bonds, thereby depleting GSH and synergistically amplifying oxidative stress, ultimately leading to TNBC cell death [101]. Curcio et al. [102] demonstrated that smart lipid-polysaccharide nanoparticles (PHYNs) have a dual design sensitive to acidic pH and tumor redox state, allowing a controlled and efficient release of doxorubicin in breast cancer cells. In the presence of high concentrations of GSH, characteristic of the tumor microenvironment, disulfide bonds are broken, facilitating drug release. Conversely, imine bonds are degraded under acidic conditions, further enhancing the effect. This induces minimal release under physiological conditions (≤33% at pH 7.4), but very high under simulated intracellular conditions (≥80%), increasing cytotoxicity against tumor cells while maintaining stability and safety in non-cancer cells.

Together, these approaches suggest that integrating biomarkers of mitochondrial dependence and oxidative stress will be key to bringing these therapies to the clinic. Furthermore, redox nanomedicine approaches, such as diselenide-based prodrugs that increase ROS and deplete GSH in TNBC cells, or redox potential-responsive nanogels that enhance doxorubicin delivery, represent a step toward more targeted therapies. Thus, these studies suggest that TNBC’s reliance on GSH and mitochondrial redox buffering can be exploited therapeutically. However, translation to the clinic remains limited by systemic toxicity, metabolic redundancy, and the tumor’s ability to rewire redox pathways. A significant gap is the absence of predictive biomarkers to stratify patients who might benefit from GSH depletion, NOX modulation, or mitochondrial inhibitors. Another limitation is that most evidence comes from cell lines or xenografts, which may not capture TNBC’s full heterogeneity. Future work should prioritize integrative approaches combining redox profiling with metabolic subtyping to guide personalized therapy.

### 11.3. Copper-Dependent Metabolism and OXPHOS Vulnerability

#### 11.3.1. Damage Caused by Copper Overaccumulation in TNBC

The importance of copper in the mitochondria of TNBC lies in the fact that several enzymes and coenzymes utilize copper as a cofactor, including cytochrome c oxidase (complex IV), and that it also regulates angiogenic mediators such as vascular endothelial growth factor (VEGF) and angiogenin [116,117]. For instance, in TNBC, the P-type copper A and B ATPase pumps (ATP7A and ATP7B), which encode copper transporters such as the copper-transporting enzyme (CTR1), are upregulated [118]. The latter impairs copper efflux, leading to intracellular copper accumulation. This increases ROS production because copper catalyzes Fenton chemistry, thereby further sustaining tumor progression [119]. However, decreased ATP7A and ATP7B have been associated with reduced tumor growth. Copper metabolism in TNBC highlights a vulnerability in redox homeostasis, but significant uncertainties remain. First, it is unclear whether copper accumulation drives TNBC progression or is a byproduct of metabolic reprogramming. Second, systemic copper modulation could affect normal tissues, given its essential role in mitochondrial enzymes and antioxidant defenses such as SOD1. This raises translational challenges, since global copper depletion risks toxicity in non-tumor tissues. A key unmet need is the identification of biomarkers that define which TNBC subtypes are most dependent on copper metabolism and would therefore respond to copper-modulating therapies (Figure 4).

To address these limitations, recent efforts have focused on nanoparticle-based copper chelators that selectively target mitochondria in TNBC, minimizing systemic effects. The next subsection examines the potential of copper-depleting nanoparticles (CDNs) as a refined therapeutic approach.

#### 11.3.2. Copper Depleting Nanoparticle (CDN) in TNBC

Cui et al. [103] have recently worked on the development of a copper-depleting nanoparticle (CDN). CDNs carry a positive charge, enabling mitochondria targeting due to the hyperpolarized mitochondrial membrane potential. Inside mitochondria, CDNs bind Cu(I) and Cu(II) and efficiently deplete intracellular copper pools. Some of the advantages of CDNs are that they are more efficient at capturing copper compared to other copper chelators, such as tris[(2-pyridyl)-methylamine (TPA) and the choline salt ATN224. CDNs are also less toxic to healthy cells than other chelators, as demonstrated by this research group, which incubated CDNs for 24 h in both healthy and TNBC cells. The results showed 60% viability in healthy cells, compared with 14% in MDA-MB-231 TNBC cells, indicating greater survival in healthy cells than in TNBC cancer cells [103].

This group also investigated whether CDNs interfered with mitochondrial function by treating breast cancer cells with CDNs. The results showed that the membrane potential decreased because of the depletion of complex IV activity. A 49% decrease in complex IV compared to the control was also observed in MDA-MB-231 cells [103]. This decrease is much greater with CDNs than with other chelators, such as TPA, because they also remove copper stores from the cytosol more efficiently. This research group also demonstrated that CDN treatment negatively regulates the transcription of complex IV subunits and chaperone proteins involved in copper transport to the mitochondria, thereby decreasing mitochondrial copper levels [103]. In this way, reduced copper levels affect mitochondrial metabolism in TNBC. It should be noted that SOD1 uses copper as a cofactor, so depleting mitochondrial copper via CDN could affect the cytosol by inactivating SOD1, thereby generating an imbalance in the mitochondrial redox state. This implies the accumulation of superoxide, which increases oxidative stress and leads to cell damage, including lipid peroxidation, a feature of ferroptosis. CDN depletes complex IV by reducing copper, an essential cofactor of this complex, which has a catalytic core containing three copper atoms. Thus, the presence of copper is vital for the assembly of the complex. A study by Ramchandani et al. [104] in TNBC cell lines showed that tetrathiomolybdate (TM), an oral copper-chelating agent, destabilizes complex IV by depleting copper, leading to its degradation and, consequently, decreasing OXPHOS. Another reported effect was that TM administration induces negative changes in mitochondrial cristae, affecting the functioning of the ETC.

Additionally, TM increases glucose consumption and activates AMP-activated protein kinase (AMPK), which, in turn, inhibits the mTOR complex 1 (mTORC1) and the acetyl-CoA carboxylase (ACC) enzyme. Both mTORC1 and ACC, which are essential for protein translation and fatty acid synthesis, respectively, are therefore inhibited, thereby limiting cancer cell growth [104]. In this way, copper metabolism not only affects mitochondrial metabolism but also protein and fatty acid metabolism.

At the mechanistic level, copper depletion disrupts several mitochondrial and redox-dependent processes essential for TNBC survival. As mentioned copper is a critical cofactor for complex IV, and its depletion impairs mitochondrial respiration, leading to reduced ATP production and increased electron leakage. This dysfunction enhances mtROS accumulation and compromises redox homeostasis. In parallel, copper depletion affects cytosolic and mitochondrial antioxidant systems, including Cu/Zn superoxide dismutase (SOD1), further limiting the cell’s capacity to detoxify ROS. Copper-depleting nanoparticles exacerbate these effects by locally reducing bioavailable copper within tumor tissue, thereby amplifying oxidative stress while minimizing systemic copper depletion. Collectively, these biochemical alterations converge on mitochondrial dysfunction and redox imbalance, sensitizing TNBC cells to cell death.

CDNs represent an innovative strategy to exploit TNBC’s copper dependency while reducing systemic toxicity. However, key translational barriers persist. First, the long-term effects of mitochondrial copper depletion on non-tumor tissues remain poorly understood, especially given copper’s essential role in neuronal and cardiac metabolism. Second, while in vitro and xenograft data are promising, there is no clinical evidence yet supporting the safety or efficacy of CDNs in humans. Finally, it remains unclear whether all TNBC subtypes are equally vulnerable to copper depletion or whether this approach is most effective in metabolically OXPHOS-dependent tumors. Addressing these questions through biomarker-driven clinical studies will be critical before advancing CDNs toward therapeutic application.

## 12. Emerging Redox-Linked Cell-Death Pathways: Cuproptosis and Disulfidptosis

Recent work has expanded the spectrum of regulated cell death mechanisms directly linked to mitochondrial metabolism and redox homeostasis, identifying cuproptosis and disulfidptosis as metabolically constrained vulnerabilities. Cuproptosis is a copper-dependent form of cell death triggered by the direct binding of copper to lipoylated components of the TCA cycle, such as dihydrolipoamide S-acetyltransferase (DLAT), leading to mitochondrial proteotoxic stress, loss of iron–sulfur cluster proteins, and collapse of oxidative metabolism [120]. This mechanism is selectively engaged in cells with active mitochondrial respiration and intact TCA cycle flux, highlighting mitochondrial metabolic state as a key determinant of sensitivity.

Although cuproptosis has not yet been systematically characterized in TNBC patient samples, its metabolic prerequisites, elevated TCA-cycle activity, reliance on oxidative phosphorylation, and copper handling, overlap with features reported in subsets of TNBC models displaying enhanced mitochondrial metabolism and resistance to glycolysis-targeted therapies [121]. This positions cuproptosis as a mechanistically relevant extension of mitochondrial redox vulnerability rather than a distinct or unrelated phenomenon.

Disulfidptosis represents a distinct redox-driven cell death mechanism induced by disulfide stress under conditions of excessive cystine uptake via SLC7A11 and insufficient NADPH-dependent reducing capacity. In this context, accumulation of intracellular disulfides leads to aberrant disulfide bond formation within actin cytoskeletal proteins, causing cytoskeletal collapse and cell death [122]. Importantly, the metabolic features that predispose cells to disulfidptosis, namely, high SLC7A11 expression, reliance on glucose-derived NADPH, and vulnerability to redox imbalance under nutrient stress, overlap with metabolic programs previously described in ferroptosis-resistant cancer cells [95,123]. In TNBC, elevated SLC7A11 expression and enhanced NADPH-dependent antioxidant capacity have been associated with resistance to lipid peroxidation–driven cell death, suggesting a potential conceptual intersection between ferroptosis resistance and susceptibility to disulfide stress, although this link has not yet been directly demonstrated in TNBC models [124,125].

Together, cuproptosis and disulfidptosis expand the conceptual framework of redox-regulated cell death beyond lipid peroxidation and caspase activation. Rather than replacing ferroptosis or apoptosis, these pathways appear to operate in parallel, with their engagement dictated by mitochondrial metabolic flux, copper availability, cystine import, and redox buffering capacity. Their inclusion underscores the need to consider mitochondrial metabolic state as a central determinant of cell-death susceptibility in TNBC.

Beyond copper-targeting strategies, other redox-related pathways, such as LOX-1 signaling, also regulate mitochondrial function, angiogenesis, and inflammatory responses in TNBC. The following subsection explores how LOX-1 blockade and targeted drug delivery can be harnessed for therapeutic benefit.

## 13. Lectin-like Oxidized LDL Receptor 1 (LOX-1) in TNBC

LOX-1 signaling has been increasingly linked to redox imbalance, mitochondrial dysfunction, and inflammatory metabolic rewiring in cancer cells, providing a rationale for its inclusion within the mitochondrial–redox framework discussed in this review.

Recently, De Vita et al. [126] developed a system in which the antineoplastic epirubicin was loaded into liposomes conjugated to LOX antibodies (LIPO-EPI-LOX). In this study, the cytotoxicity of LIPO-EPI-LOX was evaluated in MDA-MB-231 TNBC cells cultured in two-dimensional (2D) and 3D, resulting in 85% and 80% reductions in viability, respectively. This result indicates that the epirubicin’s cytotoxic remains intact in both 2D and 3D cultures. This group also evaluated the biodistribution of LIPO-EPI-LOX. Their results showed that in mice treated with LIPO-EPI-LOX, epirubicin accumulation was lower in healthy tissues such as the liver and lungs and higher in tumor tissue. The latter demonstrates that these liposomes have the potential to deliver the drug to localized sites, reducing systemic damage [126]. The study evaluated the effect of liposomes on TNBC tumors in mouse models, demonstrating that LIPO-EPI-LOX decreased tumor growth and led to 60% of cells being necrotic. This percentage was much higher than that observed with free epirubicin, which showed only 20% necrotic cells. De Vita et al. [126] also demonstrated that LIPO-EPI-LOX does not damage cardiac cells, making it a much safer therapy than the currently used free epirubicin.

It has been shown that LOX-1 overexpression promotes chemotaxis, increases inflammatory signals by increasing interleukin-8 (IL-8), activates hypoxia pathways, and increases VEGF and HIF-1α, promoting angiogenesis and tumor development [127]. In TNBC, LOX-1 is upregulated in 70% of tumor tissue, thereby favoring tumor development. Therefore, treatments that use anti-LOX antibodies would inhibit LOX-1, thereby inhibiting VEGF and HIF1α and preventing angiogenesis and cancer cell proliferation. Thus, LOX-1 inhibition may simultaneously enhance chemotherapy efficacy and disrupt tumor-promoting microenvironmental cues.

These findings highlight LOX-1 as both a biomarker and a therapeutic target in TNBC. However, questions remain regarding its specificity: LOX-1 is also expressed in endothelial and immune cells, raising concerns about potential off-target effects of LOX-1-directed therapies. Moreover, while preclinical data suggest reduced cardiotoxicity with LIPO-EPI-LOX, this needs validation in clinical trials, as long-term anthracycline-related toxicity is multifactorial. Another limitation is the absence of comparative data across TNBC subtypes; it is unclear whether LOX-1 targeting is universally effective or preferentially beneficial in highly angiogenic tumors. Addressing these gaps will be essential for translation into clinical oncology.

In addition to liposome-mediated targeting, metal-based therapeutics represent another frontier in redox modulation. The following subsection examines gold complexes and their potential to disrupt mitochondrial function in TNBC.

## 14. Gold and Mitochondria in TNBC

Gold complexes have attracted attention for their anticancer effects, particularly due to their ability to target mitochondria. Olelewe et al. [105] evaluated a cyclometallated gold(III) complex, AuPhos-19, which is lipophilic and cationic, allowing it to cross membranes and accumulate effectively in cells, particularly in mitochondria. In TNBC cell lines (MDA-MB-468, MDA-MB-231), Auphos-19 exhibited cytotoxicity, inhibiting proliferation by halting the cell cycle at the G1 phase and increasing intrinsic cytochrome c-mediated apoptosis [105]. Likewise, the possibility that AuPhos-19 alters OXPHOS was assessed by measuring oxygen consumption rate (OCR) after administration of the gold complex. The results showed a decrease in OCR, indicating that OXPHOS is inhibited, thereby reducing ATP levels. Other reported effects included activation of AMPK, an energy sensor that is activated when ATP levels are low; AMPK inhibits mTOR and ACC, reducing protein synthesis and fatty acid metabolism. In addition, AuPhos-19 can increase mtROS by depolarizing mitochondria and decrease mitochondrial DNA copies, thereby altering mitochondrial function [105]. Collectively, these effects demonstrate that AuPhos-19 alters both energy metabolism and redox state, driving TNBC cells toward apoptosis.

Gold complexes such as AuPhos-19 exhibit dual mechanisms of action: direct mitochondrial damage and modulation of redox homeostasis. However, key challenges remain. First, their selectivity for tumor versus normal cells has not been fully established, raising concerns about systemic toxicity. Second, while mitochondrial disruption appears effective in vitro, TNBC’s metabolic plasticity could enable adaptation through glycolysis or alternative metabolic programs in vivo. Third, the stability and pharmacokinetics of gold complexes in biological systems are not well characterized, which limits their clinical translation. Future research should focus on optimizing delivery systems for gold complexes, ideally in combination with metabolic inhibitors, to overcome resistance mechanisms. Despite their therapeutic promise, strategies that globally perturb redox homeostasis, such as copper depletion, NOX modulation, or metal-based mitochondrial inhibitors, raise legitimate concerns regarding toxicity to normal tissues, particularly under chronic exposure. Copper chelators, including TM, may impair essential cuproenzymes involved in mitochondrial respiration and antioxidant defense in non-malignant tissues, leading to hematological, hepatic, or neurological adverse effects [116,128,129]. Similarly, systemic inhibition of NADPH oxidases may disrupt physiological ROS signaling required for vascular tone, immune responses, and wound healing [130].

Gold-based mitochondrial inhibitors have demonstrated preferential accumulation in cancer cells due to elevated mitochondrial membrane potential; however, off-target mitochondrial toxicity remains a concern, particularly in metabolically active tissues such as heart and skeletal muscle [131,132]. These limitations underscore the narrow therapeutic window of redox-modulating agents when administered systemically.

Several strategies may enhance tumor specificity and mitigate toxicity. These include nanoparticle-based delivery systems that exploit enhanced permeability and retention effects, pro-drug designs activated by tumor-specific redox conditions, and combination approaches that allow dose reduction of individual agents [133,134]. In addition, patient stratification based on metabolic or redox biomarkers may help identify TNBC subsets more likely to respond at lower, less toxic doses.

Overall, careful consideration of redox dependency, tissue-specific vulnerabilities, and delivery strategies will be essential for the safe clinical translation of mitochondrial and redox-targeted therapies in TNBC.

Beyond inorganic complexes, innovative peptides such as GK-1 represent another redox-modulating strategy, exerting pro-oxidant effects and altering mitochondrial function in TNBC. The following subsection reviews preclinical evidence supporting GK-1 as a potential therapeutic agent.

## 15. Pro-Oxidant Effect of GK-1 in TNBC

Beyond classical redox-active compounds, immunomodulatory peptides such as GK-1 have emerged as indirect regulators of mitochondrial and redox homeostasis in TNBC. GK-1 target mitochondria and its ability to modulate inflammatory signaling, oxidative stress responses, and tumor–host interactions place it within the broader mitochondrial–redox vulnerability landscape discussed in this review. In TNBC, where chronic inflammation and redox imbalance contribute to metabolic adaptation and therapeutic resistance, GK-1 represents an example of how immune-driven redox modulation may influence mitochondrial function and tumor progression.

The immunomodulatory peptide GK-1 has been tested in TNBC xenotransplant models to evaluate its pro-oxidant effects and impact on mitochondrial metabolism [106]. In this study, it was demonstrated that GK-1 reduces catalase activity and, consequently, increases mitochondrial H_2_O_2_ accumulation. This increase in H_2_O_2_ induces oxidative stress, leading to oxidative damage, decreased membrane potential, reduced respiration, and decreased ATP production, ultimately resulting in mitochondrial dysfunction. These results demonstrate that tumor development and proliferation are limited once mitochondrial dysfunction is compromised. Therefore, GK-1 is a peptide that could be used to treat TNBC [106]. Hernandez-Aceves et al. [135] further reported that GK-1 exhibits marked antitumor activity when administered intravenously or intranasally. Treatment reduced tumor growth and modulated the tumor microenvironment without inducing significant adverse effects in critical organs, such as the heart and kidneys. Moreover, intravenous and intranasal administration were found to be safe, confirming the absence of histological and functional toxicity in these organs. This is essential when considering systemic therapies for TNBC, a highly aggressive subtype with few effective therapeutic options. This safety profile, associated with effects that reduce the invasiveness of the treatment without compromising its efficacy, combined with its ability to induce antitumor responses, positions GK-1 as an excellent innovative redox strategy, capable of offering significant therapeutic benefits without compromising the integrity of sensitive tissues, representing an advantage over conventional treatments that are often limited by their side effects.

GK-1 represents an innovative approach that combines direct redox modulation with immunomodulatory effects, positioning it as a dual-action therapeutic candidate. However, several challenges remain before translation to the clinic. First, the precise mechanism linking GK-1’s pro-oxidant activity to its immunomodulatory effects remains unclear. Second, while animal studies suggest safety, human data are entirely lacking, and it remains unclear whether chronic administration would maintain this safety profile. Third, the heterogeneity of TNBC raises questions about whether GK-1 efficacy is universal or restricted to specific subtypes or tumor microenvironments. Addressing these issues through mechanistic studies and early-phase clinical trials will be critical for determining GK-1’s true therapeutic potential.

Triple-negative breast cancer (TNBC) is characterized by pronounced metabolic plasticity, allowing tumor cells to adaptively shift between glycolysis and oxidative phosphorylation (OXPHOS) in response to environmental stress, nutrient availability, and therapeutic pressure [7,24]. This metabolic flexibility has emerged as a fundamental feature of TNBC heterogeneity and may have important implications for the response to redox-targeted therapies.

Redox-based therapeutic strategies (including mitochondrial disruptors, copper-modulating agents, and pro-oxidant approaches) aim to induce mitochondrial stress or overwhelm cellular redox buffering capacity. However, extensive evidence from cancer metabolism studies indicates that suppression of one metabolic axis can promote compensatory activation of alternative pathways, such as increased glycolytic flux or enhanced antioxidant capacity, thereby preserving redox homeostasis and cell survival [24,136].

Although direct experimental evidence linking metabolic switching to resistance against specific redox-targeted therapies in TNBC remains limited, these adaptive responses provide a plausible mechanistic basis for therapeutic escape. TNBC cells capable of dynamically reprogramming energy production and redox balance may therefore exhibit reduced sensitivity to mitochondrial or pro-oxidant stress when such therapies are applied as monotherapies.

These considerations support the rationale for combination strategies designed to restrict metabolic adaptability, such as concurrently targeting mitochondrial function and glycolytic pathways or pairing redox-inducing agents with inhibitors of antioxidant systems. While such approaches remain largely preclinical, integrating metabolic plasticity into therapeutic design may be essential for improving the efficacy of redox-based interventions in TNBC. Building on preclinical advances, several redox-targeted strategies have already progressed to clinical evaluation in TNBC patients, including vitamin C, ARQ 761, and TM. The following subsection explores these translational efforts and the challenges of moving redox modulation from bench to bedside.

## 16. Limitations of Experimental Models for Studying Mitochondrial and Redox Vulnerabilities in TNBC

Interpretation of mitochondrial and redox dependencies in TNBC is strongly influenced by the experimental model employed. Many conclusions regarding OXPHOS reliance, ROS sensitivity, or metabolic inflexibility derive predominantly from 2D cell culture systems, which do not fully recapitulate the metabolic, spatial, and redox constraints present in tumors in vivo.

In 2D cultures, TNBC cells are typically exposed to supraphysiological glucose, glutamine, and oxygen levels, conditions that artificially favor glycolysis and reduce mitochondrial stress. Under these settings, mitochondrial ROS production and redox buffering capacity may be underestimated, potentially exaggerating sensitivity to mitochondrial inhibitors or pro-oxidant therapies [137,138].

3D spheroids and organoid models partially overcome these limitations by introducing gradients of oxygen, nutrients, and metabolites. These gradients generate hypoxic and nutrient-deprived zones that promote OXPHOS rewiring, altered NAD+/NADH ratios, increased reliance on antioxidant systems, and enhanced resistance to redox-based therapies [139]. Consequently, redox vulnerabilities identified in 2D systems may not translate directly to 3D contexts.

Patient-derived xenografts (PDXs) provide additional physiological relevance by preserving tumor architecture and metabolic heterogeneity. However, species-specific differences in stromal metabolism, immune interactions, and nutrient availability limit their capacity to fully model human tumor redox biology. Moreover, pharmacokinetic and toxicity profiles of mitochondria-targeted agents often differ substantially between murine models and patients, complicating clinical extrapolation [140].

Importantly, emerging evidence indicates that TNBC metabolic states are highly plastic and context-dependent, shaped by oxygenation, stromal interactions, and therapy-induced stress. Failure to account for these model-dependent variables may lead to oversimplified conclusions regarding mitochondrial vulnerabilities or therapeutic efficacy.

Therefore, integration of multiple model systems—ranging from 2D cultures to organoids, PDXs, and metabolically characterized patient samples—will be essential to validate mitochondrial and redox targets and to improve translational relevance in TNBC.

## 17. Future Clinical Applications of Redox Therapy in TNBC

Despite the robust body of preclinical evidence supporting mitochondrial and redox vulnerabilities in TNBC, important limitations must be acknowledged regarding the predictive value of commonly used experimental models. Most mechanistic insights derive from 2D cell cultures, which are exposed to supraphysiological glucose, oxygen, and nutrient levels. These conditions tend to exaggerate glycolytic phenotypes and may underestimate mitochondrial dependence and redox constraints present in vivo. Murine xenograft models partially overcome these limitations by incorporating systemic metabolism and tumor–host interactions; however, they still fail to fully recapitulate human tumor heterogeneity, immune contexture, and metabolic diversity. Furthermore, differences in nutrient availability, copper and iron homeostasis, and antioxidant capacity between mice and humans complicate the extrapolation of redox-based therapeutic responses.

Emerging platforms such as three-dimensional cultures, patient-derived organoids, and patient-derived xenografts (PDX) offer improved preservation of tumor architecture, metabolic gradients, and subtype-specific features. These models have begun to reveal metabolic dependencies and redox adaptations not captured in conventional systems. Integrating such models with metabolic profiling and, where feasible, co-clinical trial designs may substantially improve translational accuracy and patient stratification for mitochondrial- and redox-targeted therapies in TNBC.

The next section discusses the prospects of redox therapies, with an emphasis on their current clinical development, key challenges, safety profile, and opportunities for eventual integration into translational oncology in TNBC.

## 18. Vitamin C Augments the Therapeutic Effect of Gemcitabine–Carboplatin in Advanced TNBC Patients

Pharmacological doses of vitamin C (ascorbate) have been studied as a pro-oxidant therapy for TNBC. A retrospective analysis of 70 patients with advanced TNBC evaluated the impact of adding intravenous vitamin C to standard gemcitabine and carboplatin treatment [141]. Thirty-five patients received vitamin C along with chemotherapy, while 35 served as the control group, matched by age, menopausal status, and metastatic sites. In the vitamin C group, the objective response rate was 48. 6%, compared to 40% in the non-vitamin C group (Table 5).

Additionally, median progression-free survival was 7 months versus 4.5 months, and median overall survival was 27 months versus 18 months, demonstrating significant differences. After two treatment cycles, tumor marker levels dropped substantially in the vitamin C group, and patients’ performance status, measured with the Karnofsky score, improved significantly (Table 5) [141]. Although both groups experienced typical chemotherapy side effects, the incidence of adverse reactions was lower among patients receiving vitamin C, suggesting that vitamin C may enhance treatment tolerance and quality of life, in addition to boosting chemotherapy’s effectiveness in patients with advanced TNBC [141]. It is important to note that the mechanism by which intravenous vitamin C synergizes with antineoplastic agents such as gemcitabine and carboplatin may be linked to its pro-oxidant activity at pharmacological doses. Unlike its traditional antioxidant role, high concentrations of vitamin C can produce H_2_O_2_ and other ROS in the tumor microenvironment [141]. This ROS production can overcome TNBC cells’ antioxidant defenses, inducing selective oxidative stress that may lead to apoptosis, necrosis, or ferroptosis [142,143]. Conversely, normal cells, with more robust antioxidant systems, are better able to withstand increased ROS levels. Therefore, vitamin C not only increases TNBC cells’ susceptibility to oxidative damage induced by platinum and gemcitabine but also helps explain the observed synergy, resulting in improved survival and treatment tolerance in patients [144,145].

Although vitamin C has long been viewed as a promising adjuvant, clinical evidence in TNBC remains preliminary. Limitations include variability in dosing protocols, short treatment durations, and inconsistent outcome measures across studies. Additionally, relying on intravenous delivery creates logistical challenges. A deeper mechanistic understanding of how pharmacological vitamin C interacts with TNBC subtypes and whether biomarkers can predict response will be crucial for advancing this approach.

**Table 5:** Treatment of TNBC associated with redox therapy and mitochondrial metabolism in the clinical context.

Treatment	Results	Reference
Vitamin C + gemcitabine + carboplatin	Patients who received vitamin C in addition to the chemotherapy group significantly improved their condition as assessed by the Karnofsky score.	[141]
ARQ 761	ARQ 761 is activated in tumors with overexpression of the NQO1 enzyme, generating overproduction of ROS, causing necrotic cell death in a phase I trial in patients with refractory advanced/metastatic TNBC solid tumors.	[146]
TM	TM reduces copper levels, decreasing collagen remodeling, OXPHOS, and the metastasis tumor microenvironment in TNBC patients.	[147]

## 19. ARQ 761 Generates ROS Overproduction via NQO1 in TNBC Patients

NQO1 is overexpressed in several solid tumors, including TNBC [148]. This quality confers TNBC intrinsic resistance to currently available cancer therapies; however, it may also serve as a therapeutic target for redox modulation. For instance, Gerber et al. [146] demonstrated that ARQ 761, an analogue of β-lapachone, administered intravenously in a stepped dose schedule, is activated in tumors with overexpression of the NQO1 enzyme, generating ROS overproduction and programmed necrotic cell death in a phase I trial involving patients with refractory advanced/metastatic solid tumors. Although complete responses were not reported, disease stabilization was observed in several patients, especially those with elevated NQO1 expression, among whom prolonged clinical benefit was observed. The mechanism of action of ARQ172 is associated with a futile redox cycle in NQO1+ cancer cells, where NQO1 reduces ARQ 761 to an unstable hydroquinone, which is autoxidized to quinone, generating O_2_ and H_2_O_2_ in the process. In addition to the previously produced ROS, NQO1 consumes NADPH at a high rate, depleting the cell’s reducing capacity [149]. Therefore, although NQO1 is an enzyme that protects against ROS, when it encounters ARQ761, it becomes a “Trojan horse” that generates excess ROS, leading to the formation of unstable hydroquinone. Therefore, when a cell has an excess of this enzyme, more ROS are generated, which is why it was used in the previous clinical study.

ARQ 761 is one of the most mechanistically rational redox therapies, leveraging a tumor-specific vulnerability (NQO1 overexpression). However, clinical application faces challenges: variability of NQO1 expression among TNBC tumors, potential off-target effects in normal tissues with basal NQO1 activity, and dose-limiting toxicities observed in early trials. Identifying reliable biomarkers to stratify patients by NQO1 status will be crucial for clinical success.

## 20. TM Lowers Copper Levels, Reducing Collagen Remodeling, OXPHOS, and the Metastatic Tumor Microenvironment in TNBC Patients

Interestingly, a phase II clinical trial in 36 patients with TNBC at high risk of relapse (including stages II, III, and IV) demonstrated that TM reduced endothelial progenitor cells (EPCs), which play a key role in shaping the metastatic tumor microenvironment, and decreased LOXL-2 levels, indicating a favorable modification of the tumor microenvironment [147]. Furthermore, this study showed a low relapse rate in this high-risk population, with patients remaining disease-free at 6.3 years, achieving an event-free survival (EFS) of 90% in stage II/III TNBC and 50% in stage IV with no evidence of disease (NED). This study also demonstrated that TM was well tolerated, with severe toxicities observed in only a few patients. Reversible neutropenia was observed in 2–3% of patients, and rare cases of anemia or fever were reported [147]. Derived from these results in patients, the authors of the previous study hypothesized that TM causes global changes in the tumor and host microenvironment, making it inhospitable to tumor progression, and consequently performed studies associated with the extracellular matrix (ECM), which is crucial for interactions between cancer and stromal cells, allowing the promotion of invasion and metastasis [150]. One of the key proteins for the ECM is collagen which provides the structural and molecular framework for tumor progression by facilitating focal adhesion, cell proliferation and motility, and the availability of copper to be mobilized from existing stores, finding that TM decreased collagen in murine tumor models, in these models they also found that TM suppresses lung metastases and LOX levels and activity [147,151]. The above suggests that TM, in addition to depleting copper and LOXL2 in EPCs, may be involved in a series of downstream effects that potentially render the tumor microenvironment suboptimal for metastasis progression, thereby promoting the motion of tumor dormancy.

Despite a promising rationale, TM encounters translational challenges. Copper is vital for normal physiology, and long-term depletion can cause anemia, leukopenia, or immunosuppression. Additionally, the therapeutic window for effective copper depletion without causing systemic toxicity is narrow. Clinical evidence in TNBC remains limited, and combination regimens will likely be necessary to achieve sustained benefits.

Overall, these clinical efforts with vitamin C, ARQ 761, and TM show the potential of redox-based therapies. However, they also reveal ongoing challenges: identifying predictive biomarkers, managing systemic toxicity, and tailoring treatments to TNBC heterogeneity. These lessons emphasize the importance of future studies that not only test new agents but also incorporate metabolic and redox profiling into clinical trial design.

Although redox modulation has shown promising results in preclinical studies, including cell and murine models, its translation into clinical practice is still in its infancy. The development of therapies based on redox manipulation faces significant limitations, including the complexity of redox signaling networks in heterogeneous tumor microenvironments, interindividual variability in response, and issues related to pharmacokinetics, bioavailability, and off-target toxicity. However, several redox-modulating agents are already in early or intermediate clinical evaluation, providing valuable insights into their potential and limitations. For instance, the possibility of combining disulfiram (DSF), an aldehyde dehydrogenase inhibitor with pro-oxidant properties, with copper has also been explored. An ongoing clinical trial (NCT03323346) is evaluating this strategy in patients with recurrent or metastatic breast cancer in general, without restricting it to the TNBC subtype. However, no results have been published to date. Since it was not explicitly designed for TNBC and not reported in subanalyses by subtype, the available evidence remains insufficient to establish its clinical relevance in this context.

Given the pronounced metabolic and redox heterogeneity of TNBC, patient stratification is likely to be essential for the success of these approaches. Several classes of biomarkers may be relevant in this context. First, metabolic gene-expression signatures reflecting mitochondrial activity, such as enrichment of OXPHOS or PGC-1α–associated transcriptional programs, have been linked to mitochondrial dependence and therapeutic vulnerability in breast cancer models [152]. Second, redox-related enzymes and transporters (including components of the GSH and thioredoxin systems) may inform sensitivity to pro-oxidant or antioxidant-targeting therapies, as these pathways critically regulate intracellular redox buffering capacity [85,95].

In addition, specific enzymatic dependencies offer more direct biomarker opportunities. For example, overexpression of NQO1 has been exploited therapeutically using β-lapachone derivatives, where NQO1 levels predict tumor-selective ROS generation and cytotoxicity [146]. Similarly, markers of mitochondrial function, such as OCR, mitochondrial mass, or expression of electron transport chain components, may help identify TNBC subsets with heightened sensitivity to mitochondrial stress.

Importantly, most candidate biomarkers have been defined in preclinical settings, and their validation in patient-derived models and clinical trials remains limited. Future studies integrating metabolic profiling, redox enzyme expression, and TNBC molecular subtyping will be critical for translating redox-based therapies into precision oncology strategies.

This review emphasizes the advantages, limitations, and development stages (preclinical versus clinical) of each redox approach (GSH depletion, NOX modulation, copper depletion, gold complexes, etc.), along with several considerations outlined in Table 6. Concerning the latter, most studies using cellular models rely on established TNBC lines, which, while helpful in understanding basic mechanisms, do not fully capture the genetic and metabolic diversity observed in human tumors. Furthermore, although murine models help integrate tumor processes and systemic responses, physiological differences from humans limit the direct application of these findings. Lastly, evidence from clinical trials involving TNBC patients remains limited, heterogeneous, and often based on small sample sizes, which complicates drawing definitive conclusions about the potential of redox-targeted therapies. Overall, these limitations highlight the need for future research that more effectively connects preclinical data with well-structured clinical studies. Despite these challenges, this review offers a comparative analysis that systematically synthesizes evidence from cellular models, animal studies, and patient data, enabling the identification of shared patterns and discrepancies in TNBC redox responses. This cross-sectional approach reveals knowledge gaps and suggests directions for future research to help translate preclinical findings into clinical practice. Additionally, by compiling and analyzing the latest insights into the role of redox status in TNBC, this review provides a conceptual framework to guide the development of new therapeutic strategies and promote the integration of fundamental biology with clinical applications.

## 21. Translational Challenges and Clinical Stratification in Targeting Mitochondrial and Redox Vulnerabilities in TNBC

Despite the growing recognition of mitochondrial metabolism and redox imbalance as therapeutic vulnerabilities in TNBC, clinical translation remains challenging. A major limitation is the lack of robust biomarkers capable of stratifying patients according to mitochondrial dependency, redox buffering capacity, or susceptibility to specific forms of regulated cell death.

Most clinical trials targeting metabolic or redox pathways have relied on histological subtype or receptor status alone, without incorporating functional metabolic profiling. However, accumulating evidence indicates that parameters such as mitochondrial respiration rates, NAD+/NADH balance, glutathione availability, and expression of antioxidant systems (e.g., SLC7A11, GPX4, NQO1) strongly influence therapeutic response and resistance [85].

Another translational barrier is the pharmacokinetic and toxicity profile of mitochondria-targeted agents. Compounds that disrupt OXPHOS or elevate mitochondrial ROS often show narrow therapeutic windows, as mitochondrial metabolism is essential for normal tissues, including cardiomyocytes, neurons, and immune cells. This limitation has been observed in early clinical experiences with mitochondrial complex I inhibitors and redox-cycling agents, where systemic toxicity constrained dosing despite promising preclinical efficacy [61,153].

Furthermore, metabolic plasticity enables TNBC cells to bypass targeted interventions through compensatory pathways, such as switching between glycolysis and OXPHOS, activating NADPH-producing routes (PPP, malic enzyme, IDH1), or enhancing cystine uptake and thiol-based redox buffering. These adaptive responses often emerge under therapeutic pressure and contribute to acquired resistance [24,136].

Importantly, the absence of standardized metabolic or redox profiling in TNBC clinical trials hampers rational patient selection. Emerging approaches, including metabolomics, functional imaging of mitochondrial activity, and ex vivo drug testing in patient-derived organoids, may offer opportunities to bridge this gap and guide combination strategies targeting dual metabolic axes [154,155].

Together, these challenges highlight the need for integrated translational frameworks combining metabolic biomarkers, subtype-specific vulnerabilities, and adaptive resistance mechanisms to enable effective clinical exploitation of mitochondrial and redox targets in TNBC.

## 22. Discussion

TNBC remains one of the most aggressive breast cancer subtypes, mainly due to the lack of well-defined molecular targets and its significant heterogeneity. Several reviews have independently examined redox signaling in oncology or TNBC metabolism, but few have integrated mitochondrial metabolism, redox regulation, and emerging therapies into a unified framework. This review aims to address this gap by connecting TNBC metabolic subtypes, their redox balance dependence, and new therapeutic opportunities. A common theme in preclinical and translational studies is that TNBC shows metabolic flexibility, switching between glycolysis and OXPHOS in response to environmental stresses and treatment pressures. Evidence indicates that OXPHOS dependency is significant in specific subtypes, where mitochondrial ROS production supports survival and metastasis. This dependence reveals a therapeutic vulnerability; however, controversy persists: some studies highlight glycolysis as the dominant pathway, while others emphasize OXPHOS as essential. The most compelling data suggest a context-dependent duality, in which targeting redox balance may be feasible only when combined with methods that inhibit compensatory pathways.

From a translational perspective, redox therapies such as vitamin C, ARQ 761, and TM illustrate both the promise and limitations of this strategy. The most substantial evidence supports exploiting tumor-specific vulnerabilities—such as NQO1 overexpression in the case of ARQ 761—rather than indiscriminately applying redox agents. Yet systemic toxicity, narrow therapeutic windows, and lack of predictive biomarkers remain significant barriers. Identifying robust biomarkers (e.g., redox gene signatures, metabolic subtype classification, mitochondrial activity markers) should be prioritized in future clinical trials to stratify patients most likely to benefit.

Another emerging insight is the potential of integrative strategies. Metal-based compounds (copper, gold), redox nanoparticles, mitochondrial-targeted molecules, and peptides such as GK-1 exhibit synergistic effects when combined with conventional or metabolic therapies. These approaches not only induce oxidative stress but also disrupt tumor-supporting pathways, including angiogenesis, fatty acid metabolism, and immune evasion. Future research should focus on rational combinations that exploit these interactions while minimizing systemic toxicity. Of course, much research remains to be performed to determine at which TNBC stages a particular treatment can be used, given that cancers are heterogeneous.

## 23. Conclusions

In conclusion, integrating redox state, mitochondrial metabolism, and therapeutic innovation offers a new perspective on addressing TNBC. While existing reviews have examined redox biology or TNBC metabolism separately, this synthesis highlights their convergence as a therapeutic opportunity. By critically evaluating the most substantial evidence, acknowledging controversies, and emphasizing unmet clinical needs, this review underscores that advancing TNBC therapy will depend on the following:

Developing biomarkers to identify redox- and metabolism-dependent TNBC subsets.

Designing therapies that block compensatory metabolic adaptations.

Prioritizing translational studies that balance efficacy with systemic safety.

This redox-centered framework provides a more nuanced understanding of TNBC biology and offers a roadmap for next-generation treatments that go beyond chemotherapy toward precision metabolic and redox medicine.

## Figures and Tables

**Table 1:** TNBC molecular subtypes and metabolic–redox features.

TNBC Subtype	Dominant Metabolic Traits	Redox Characteristics	Therapeutic Implications
BL1/BL2	High glycolysis, DDR	Elevated ROS tolerance	Pro-oxidant strategies, DNA damage enhancers
M/MSL	OXPHOS, lipid metabolism	Mitochondrial ROS reliance	Mitochondrial inhibitors, redox-active metals
LAR	Lipid & steroid metabolism	Strong antioxidant buffering	Combination metabolic–redox inhibition

**Table 2:** TNBC subtype-specific display distinct metabolic phenotypes and redox vulnerabilities, and the possible therapeutics.

TNBC Subtype	Predominant Metabolic Features	Redox Characteristics	Potential Vulnerabilities	Therapeutic Implications
BL1/BL2	High proliferation; glycolysis with inducible OXPHOS	Elevated ROS, moderate antioxidant buffering	Sensitivity to ROS overload, DNA damage	Pro-oxidant therapies; chemotherapy combinations
Mesenchymal (M)	OXPHOS reliance; fatty acid oxidation	High mitochondrial ROS; NRF2 activation	Mitochondrial stress; redox imbalance	OXPHOS inhibitors; mitochondrial disruptors
MSL	Metabolic flexibility; EMT-associated metabolism	Enhanced antioxidant programs	Resistance to redox stress	Combination metabolic + redox targeting
IM	Variable metabolism influenced by immune context	Redox modulation of immune infiltration	ROS-sensitive immune interactions	Redox-immunometabolic combinations
LAR	Lipid metabolism; mitochondrial dependence	NADPH-dependent redox buffering	FAO and mitochondrial vulnerability	Metabolic inhibitors; redox modulation
HER2-low	Partial ERBB signaling; mitochondrial engagement (indirect evidence)	Enhanced antioxidant capacity	Mitochondrial stress sensitivity	Mitochondrial targeting + ADCs (hypothesis-driven)

**Table 3:** Conflicting evidence supporting glycolytic versus OXPHOS-dependent metabolism in TNBC.

Aspect	Glycolysis-Dominant TNBC	OXPHOS-Dependent TNBC
Key metabolic features	High glucose uptake, elevated glycolytic enzymes, lactate production	Increased mitochondrial respiration, TCA cycle activity, ETC gene expression
Experimental models	Predominantly 2D cell cultures	3D cultures, PDX, in vivo models
Representative observations	Sensitivity to glycolysis inhibitors	Sensitivity to OXPHOS or mitochondrial inhibitors
Limitations	Overestimation due to high-glucose media	Often subtype- or context-dependent
Unresolved questions	Persistence in vivo?	Compensation via glycolysis?

**Table 4:** Molecules and their targets for the treatment of TNBC associated with redox therapy and mitochondrial metabolism in vivo and in vitro models.

Molecule	Target	Mechanism	Reference
SASP Erastin BSO	GSH	Decrease of GSH and γ-glutamylcysteine ligase, resulting in TNBC cell death such as ferroptosis.	[94]
NOX4 overexpression	NOX4	Overexpression of NOX4 decreased cell migration, invasiveness, and proliferation of TNBC cells. Loss of NOX4 worsens tumor prognosis, and in vitro NOX4 silencing enhanced proliferation, colony formation, and migratory capacity.	[97] [98]
CPI-613	α-ketoglutarate dehydrogenase	Increase metabolic stress in specific subtypes of TNBC cell lines.	[99]
MitoQ	Mitochondrial redox state	Prevents metastasis of TNBC murine models.	[100]
FA-SeSe-NPs	GSH	Induces ROS accumulation, which reacts with GSH, depleting GSH and synergistically amplifying oxidative stress, inducing TNBC cell death.	[101]
PHYNs	Drug release	In the presence of high concentrations of GSH, characteristic of the tumor microenvironment, disulfide bonds are broken, facilitating drug release such as doxorubicin in TNBC cells.	[102]
CDN	Mitochondrial complex IV SOD1	CDN decreases mitochondrial metabolism and deactivates SOD1, generating oxidative stress, which leads to lipid peroxidation and cell damage in TNBC cells.	[103]
TM	Mitochondrial complex IV	Destabilizes complex IV by depleting copper, leading to its degradation and decreasing OXPHOS, TM also changes in the mitochondrial cristae in a cell line of TNBC.	[104]
AuPhos-19	OXPHOS mtROS	AuPhos-19 increases intrinsic cytochrome c-mediated apoptosis in the TNBC cell line. AuPhos-19 also alters OXPHOS, causing a reduction in ATP levels. AuPhos-19 also increases mitochondrial ROS by depolarizing mitochondria, in addition to decreasing the number of mitochondrial DNA copies, which alters mitochondrial function.	[105]
GK-1	Mitochondrial metabolism mtROS	GK-1 decreases catalase enzyme activity, increasing H_2_O_2_ in mitochondria, inducing oxidative stress and oxidative damage, leading to a decrease in membrane potential and mitochondrial dysfunction, resulting in a decrease in ATP production in a TNBC xenograft.	[106]

**Table 6:** Comparative overview of redox-targeted therapeutic strategies in TNBC.

Strategy/Agent	Primary Target	Mechanism of Action	Dominant Redox Effect	TNBC Subtype Relevance	Preclinical/Clinical Status
Sulfasalazine (SASP)	SLC7A11 (xCT)	Inhibits cystine uptake, impairs GSH synthesis	↑ ROS, ferroptosis sensitization	Basal-like, mesenchymal, SLC7A11-high	Preclinical
Buthionine sulfoximine (BSO)	γ-GCS	Blocks de novo GSH synthesis	GSH depletion, ↑ oxidative stress	Basal-like TNBC	Preclinical
GK-1	Catalase/redox balance	Reduces catalase activity, promotes accumulation of H_2_O_2_	↑ intracellular ROS	Aggressive/chemoresistant TNBC	Preclinical
Vitamin C (high-dose)	Redox cycling	Pro-oxidant via H_2_O_2_ generation at pharmacologic doses	Oxidative stress overload	Glycolytic TNBC	Preclinical/early clinical
ARQ 761 (β-lapachone analog)	NQO1	NQO1-dependent redox cycling, induces NAD^+^/ATP depletion	ROS burst, energetic collapse	NQO1-high TNBC	Preclinical/clinical development
Copper depletion (TM)	Bioavailable Cu	Inhibits Cu-dependent enzymes (SOD1, Complex IV)	mtROS increase, OXPHOS disruption	OXPHOS-dependent TNBC	Clinical (repurposed)
Copper-depleting nanoparticles (CDNs)	Intracellular Cu	Selective Cu sequestration	mtROS, mitochondrial dysfunction	Mitochondria-dependent TNBC	Preclinical
Gold complexes (AuPhos-19)	Mitochondria/ETC	Disrupts ETC and thiol redox systems	mtROS induction	OXPHOS-high TNBC	Preclinical
Metformin/Phenformin	Complex I	Inhibits mitochondrial respiration	↓ ATP, compensatory ROS	OXPHOS-dependent TNBC	Clinical/preclinical
Redox nanomedicine	ROS sensing or generation	ROS-responsive or ROS-generating nanoplatforms	Context-dependent ROS modulation	Heterogeneous TNBC	Preclinical
PHYNs	GSH pool	ROS-triggered payload release	GSH depletion	Antioxidant-adapted TNBC	Preclinical

## Data Availability

No new data were created or analyzed in this study.

## Abbreviations

ER	Estrogen receptor
TNBC	Triple-negative breast cancer
PR	Progesterone receptor
HER2	Human epidermal growth factor receptor 2
ROS	Reactive oxygen species
OXPHOS	Oxidative phosphorylation
GE	Gene expression
BL1	Basal-like
IM	Immunomodulatory
M	Mesenchymal
MSL	Mesenchymal stem-like
LAR	Luminal androgen receptor
SOD	Superoxide dismutase
O_2_^−^	Superoxide oxygen
H_2_O_2_	Hydrogen peroxide
CAT	Catalase
GPx	Glutathione peroxidase
Vitamin C	Ascorbic acid
GSH	Glutathione
Vitamin E	Tocopherol
ATP	Adenosine triphosphate
ETC	Electron transport chain
NADH	Nicotinamide adenine dinucleotide
FADH2	Flavin adenine dinucleotide
Q	Coenzyme Q10
QH2	Ubiquinol
cyt C	Cytochrome C
H^+^	Protons
ADP	Adenosine diphosphate
O_2_	Oxygen
MnSOD	Mn superoxide dismutase
Cu/ZnSOD	Cu/Zn superoxide dismutase
NOX	NADPH oxidase
^•^OH	Hydroxyl radical
8-OHdG	8-hydroxy-2-deoxyguanosine
Nrf2	Nuclear factor erythroid 2-related factor 2/Erythroid 2-related factor 2
DCIS	Ductal carcinoma in situ
LCIS	Lobular carcinoma in situ
NST	Non-special type ductal carcinoma
LC	Lobular carcinoma
PARP	Poly (ADP-ribose) polymerase
BRCA	BReast CAncer gene 1
PD-L1	Programmed death-Ligand 1
MAP	Microtubule-associated protein
pCR	Pathological complete response
DFS	Disease-free survival
HIF	Hypoxia-inducible transcription factor
PKM2	Pyruvate Kinase M2
LDHA	Lactate Dehydrogenase A
NED	No evidence of disease
DSF	disulfiram
EPC	Endothelial progenitor cells
2D	Two-dimensional
3D	Three-dimensional
PI3K	Phosphoinositide 3-kinase
mTOR	Mammalian target of rapamycin
OCR	Oxygen consumption rate
IL-8	Interleukin-8
ACC	Acetyl-CoA carboxylase
mTORC1	mTOR complex 1
AMPK	AMP-activated protein kinase
TM	Tetrathiomolybdate
TPA	Tris[(2-pyridyl)-methylamine
CDN	Copper-depleting nanoparticle
CTR	Copper-transporting enzyme
VEGF	Vascular endothelial growth factor
mtROS	Mitochondrial reactive oxygen species
EMT	Epithelial-mesenchymal transition
TCA	Tricarboxylic acid
IDH1	Isocitrate dehydrogenase
OPA1	Optic atrophy protein 1
MFN 1/2	Mitofusins 1/2
DRP1	Dynamin-related protein 1
Trx	Thioredoxin
IDH1	Isocitrate dehydrogenase 1
ME1	malic enzyme 1
NADPH	Nicotinamide adenine dinucleotide phosphate
PPP	phosphate pathway
PTEN	Phosphatase and tensin homologue
KEAP1	Kelch-like ECH-associated protein 1
GLUT1	Glucose transporter type 1
PGC-1α	Peroxisome proliferator-activated receptor-gamma coactivator 1-alpha
NQO1	NAD(P)H quinone oxidoreductase 1
ADCs	Antibody drug conjugates
HMGB1	High Mobility Group Box 1
